# Protein kinase Cγ in cerebellar Purkinje cells regulates Ca^2+^-activated large-conductance K^+^ channels and motor coordination

**DOI:** 10.1073/pnas.2113336119

**Published:** 2022-02-10

**Authors:** Masashi Watanave, Nobutaka Takahashi, Nobutake Hosoi, Ayumu Konno, Hikaru Yamamoto, Hiroyuki Yasui, Mika Kawachi, Takuro Horii, Yasunori Matsuzaki, Izuho Hatada, Hirokazu Hirai

**Affiliations:** ^a^Department of Neurophysiology & Neural Repair, Gunma University Graduate School of Medicine, Maebashi, Gunma 371-8511, Japan;; ^b^Viral Vector Core, Gunma University Initiative for Advanced Research, Maebashi, Gunma 371-8511, Japan;; ^c^Laboratory of Genome Science, Biosignal Genome Resource Center, Institute for Molecular and Cellular Regulation, Gunma University, Maebashi, Gunma 371-8512, Japan

**Keywords:** motor, kinase, dendritic computation

## Abstract

The cerebellum, the site where protein kinase C (PKC) was first discovered, contains the highest amount of PKC in the central nervous system, with PKCγ being the major isoform. Systemic PKCγ-knockout (KO) mice showed impaired motor coordination and deficient pruning of surplus climbing fibers (CFs) from developing cerebellar Purkinje cells (PCs). However, the physiological significance of PKCγ in the mature cerebellum and the cause of motor incoordination remain unknown. Using adeno-associated virus vectors targeting PCs, we showed that impaired motor coordination was restored by re-expression of PKCγ in mature PKCγ-KO mouse PCs in a kinase activity–dependent manner, while normal motor coordination in mature *Prkcg^fl/fl^* mice was impaired by the Cre-dependent removal of PKCγ from PCs. Notably, the rescue or removal of PKCγ from mature PKCγ-KO or *Prkcg^fl/fl^* mice, respectively, did not affect the CF innervation profile of PCs, suggesting the presence of a mechanism distinct from multiple CF innervation of PCs for the motor defects in PKCγ-deficient mice. We found marked potentiation of Ca^2+^-activated large-conductance K^+^ (BK) channel currents in PKCγ-deficient mice, as compared to wild-type mice, which decreased the membrane resistance, resulting in attenuation of the electrical signal during the propagation and significant alterations of the complex spike waveform. These changes in PKCγ-deficient mice were restored by the rescue of PKCγ or pharmacological suppression of BK channels. Our results suggest that PKCγ is a critical regulator that negatively modulates BK currents in PCs, which significantly influences PC output from the cerebellar cortex and, eventually, motor coordination.

## Significance

The cerebellum, the site where protein kinase C (PKC) was discovered, contains the highest amount of PKCγ in the central nervous system. PKCγ in the cerebellum is exclusively confined to Purkinje cells (PCs), sole outputs from the cerebellar cortex. Systemic PKCγ-knockout mice show impaired motor coordination; however, the cause of motor defects remains unknown. Here we show that activation of PKCγ suppresses the Ca^2+^-activated large-conductance K^+^ (BK) channels located along the PC dendrites. A consequential increase in the membrane resistance attenuates electrical signal decay during propagation, resulting in an altered complex spike waveform. Our results suggest that synaptically activated PKCγ in PCs plays a critical role in motor coordination by negative modulation of BK currents.

Protein kinase C (PKC) was originally identified by the late Yasutomi Nishizuka (1, 2), also known as the father of PKC (3). In the 1970s, Nishizuka’s group used the bovine cerebellum to study the properties of cyclic guanosine monophosphate (cGMP)–dependent protein kinase. During this experiment, they identified a novel cyclic nucleotide–independent kinase (4), which required an unphysiologically high concentration of Mg^2+^ (50 to 100 mM), and it was initially named protein kinase M (PKM) (4). Subsequent studies by Nishizuka et al. proved that PKM could be activated by physiological concentrations of Ca^2+^ (less than 5 × 10^−5^ M) in the presence of phospholipids, and consequently, it was renamed as PKC (5).

The overlap of specific experimental conditions serendipitously led to the discovery of PKC, one of which would be usage of the cerebellum since this organ was later proven to contain the highest amount of PKC in the central nervous system (CNS) (6). The cerebellum expresses all subspecies of classical PKCs (cPKCs), namely PKCα, PKCβI, PKCβII, and PKCγ, among which PKCγ is a major isotype accounting for over half of the cPKCs in the cerebellum (6). Immunohistochemistry showed that PKCγ in the cerebellum is densely and exclusively confined to Purkinje cells (PCs) (7–9), and it continues to be expressed throughout life. Our previous analyses using single PC real-time reverse transcription-quantitative PCRs and Western blotting showed that PKCγ accounts for 98% and 96% of cPKC mRNA and protein, respectively, in adult mouse PCs (10). Thus, it is reasonable to assume that extremely high amounts of PKCγ expressed in PCs play significant physiological roles in PCs and, consequently, in cerebellar function.

Systemic PKCγ-knockout (KO) mice were generated in the early 1990s (11). Mutant mice show impaired motor coordination (12). Electrophysiological analysis of mature mutant mice revealed deficient pruning of climbing fiber (CF) synapses from PCs, which otherwise occurred during postnatal development, and this resulted in the innervation of PCs by supernumerary CFs even after their maturation (13). Based on the observation of PKCγ-KO mice and several other gene KO mice, it was proposed that persistent multiple CF innervations of mature PCs underlay motor incoordination (12). However, some gene-modified mice were later discovered which showed overall normal motor coordination with persistent multiple CF innervation of PCs (14, 15). Thus, the mechanism underlying motor defects in PKCγ-KO mice remains unknown. Motor incoordination may be a consequence of the persistent innervation of a PC by multiple CFs, as proposed over a quarter of a century ago (12). However, given that PKCγ is a neuron-specific isotype expressed throughout the CNS (6), motor defects could be due to unclarified developmental defects in the cerebellum or other CNS regions or due to the loss of PKCγ-regulated bioactivity in the mature mouse CNS. We attempted to answer this long-standing question using viral vector–based approaches. We rescued PKCγ or kinase-negative PKCγ exclusively in the PCs of adult PKCγ-deficient mice using viral vectors carrying the PC-specific L7 promoter (16), and determined whether the aberrant phenotypes, including multiple CF innervations of the PC and motor defects, were restored accordingly. We also used PC-specific viral vectors expressing Cre recombinase and eliminated PKCγ expression specifically from the PCs of adult *Prkcg^fl/fl^* mice, which could cause motor impairment if PKCγ served as a critical kinase or a bioactive substance in mature mouse PCs.

CF input triggers significant depolarization in PC dendrites, which, in turn, activates voltage-gated Ca^2+^ channels (VGCCs), leading to a large amount of Ca^2+^ influx. The elevation of cytoplasmic Ca^2+^ initiates Ca^2+^-dependent processes, which are tightly regulated by local Ca^2+^ concentrations. Ca^2+^-activated large-conductance K^+^ (BK) channels are expressed throughout most regions of the mammalian brain (17). The activation of BK channels requires a combination of membrane depolarization and an increase in cytoplasmic Ca^2+^ through the VGCCs, and this activation plays important physiological roles in the CNS, including repolarizing the action potential, shaping dendritic Ca^2+^ spikes, and modulating neurotransmitter release (18). In PCs, BK channels are colocalized with VGCC clusters along the dendritic shafts to the soma (19, 20), and these are activated by Na^+^ and Ca^2+^ spikes (21, 22). The extent of BK channel activation can define the complex spike shape since the CF input to the PC evokes a complex spike composed of large depolarization with dendritic Ca^2+^ spikes and a burst of Na^+^ spikes generated in the initial segment of the axon (23, 24). In this study, we show that synaptically activated PKCγ suppresses BK channels, and thus, it increases membrane resistance in PC dendrites in a kinase activity–dependent manner, which prevents electrical signal decay during propagation through PC dendrites. Such synaptic plasticity regulates the complex spike shape and, eventually, motor coordination.

## Results

### Restoration of Motor Defects by Re-expression of PKCγ in Mature PKCγ-KO Mouse PCs.

**Fig. 1.**
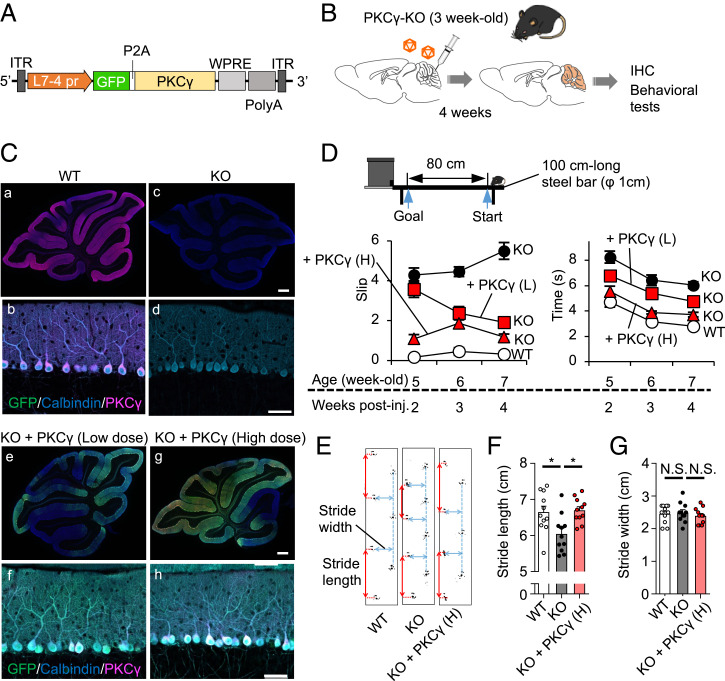
Restoration of motor defects in PKCγ-KO mice by re-expression of PKCγ in PCs. (*A*) Schema of AAV9 vectors expressing GFP-P2A-PKCγ by the PC-specific L7-4-minCMV promoter. (*B*) AAV vector-mediated PC-specific re-expression of PKCγ specifically in PKCγ-KO mice. Three-week-old PKCγ-KO mice received a cerebellar injection of the AAV9 vectors as illustrated in *A* at two different viral doses (1.6 × 10^9^ vg/mouse and 1.6 × 10^10^ vg/mouse). The cerebella of treated mice were examined by immunohistochemistry 4 wk after the viral injection. (*C*) Immunohistochemistry of the cerebellar sections from WT (*a* and *b*), naïve PKCγ-KO (*c* and *d*), and AAV-treated KO (*e* and *f*: low-dose; *g* and *h*: high-dose) mice. Sections were triple-immunostained with antibodies for GFP (green), PKCγ (magenta), and calbindin (a marker for PC) (blue). Fluorescent signals were obtained using a confocal microscope. (Scale bars, 50 μm.) (*D*) Beam-walking test. WT, PKCγ-KO, and AAV9-treated KO mice walked on a 100-cm and φ1-cm steel bar. Time spent on the 80-cm-walk (*Right* graph) and the number of slips during walking (*Left* graph) were measured. (*E*) Representative footprints from the WT (*Left*), PKCγ-KO (*Middle*), and AAV9-treated KO (*Right*) mice. (*F* and *G*) Stride lengths (red) and stride widths (blue) are summarized in *F* and *G*, respectively (10 mice in each group, *P < 0.05 Bonferroni post hoc test following one-way ANOVA). H, high dose; IHC, immunohistochemistry; ITR, inverted terminal repeat; L, low dose; PolyA, polyadenylation signal sequence; L7-4 pr, L7-4 promoter with minimal cytomegalovirus sequence; WPRE, woodchuck hepatitis virus posttranscriptional regulatory element; N.S., not significant.

Systemic PKCγ-KO mice show motor defects; however, it has not been convincingly proven that impaired motor function is attributable to the loss of PKCγ in PCs and not in different CNS regions. To verify this, we expressed PKCγ specifically in the PCs of mature PKCγ-KO mice. Adeno-associated virus serotype 9 (AAV9) vectors expressing enhanced green fluorescent protein (GFP)-porcine teschovirus-1–derived 2A peptide (P2A)-PKCγ under the control of a PC-specific L7-4 promoter fused with minimal cytomegalovirus promoter sequence (L7-4-minCMV promoter) (16) with two different viral titers (1.6 × 10^9^ or 1.6 × 10^10^ viral genome (vg)/mouse) were injected into the cerebellar cortex of postnatal day (P) 21 to 25 PKCγ-KO mice (Fig. 1 *A* and *B*). Four weeks after viral injection, sagittal sections of the cerebellum were produced and triple-immunolabeled for GFP, PKCγ, and calbindin D28K (a marker for PCs). Confocal laser-scanning microscopy showed the presence and absence of PKCγ expression in PCs of wild-type (WT) and PKCγ-KO mice, respectively (Fig. 1 *C*, *a*–*d*). In rescue mice, PKCγ and GFP were expressed specifically in PCs in a viral titer–dependent manner (Fig. 1*C*, *e*–*h*).

To examine motor function, we used a horizontal thin rod (beam)-walking test (schema in Fig. 1*D*) as previously used for the behavioral assessment of PKCγ-KO mice (12). We confirmed significantly poorer performance in 5- to 7-wk-old PKCγ-KO mice compared to age-matched WT mice, which could walk smoothly to the shelter located at the opposite end of the bar with almost no slipping, whereas the PKCγ-KO mice wobbled on the bar with frequent slips, resulting in a significantly longer duration to reach the shelter. AAV vector–mediated rescue of PKCγ in PKCγ-KO mouse PCs significantly improved their performance (fewer slips and shorter time to reach the shelter) in a viral titer–dependent manner, with restoration consistently observed during the observation period (2 to 4 wk postinjection) (graphs in Fig. 1*D* and Movie S1). Walking performance was evaluated using footprints of these mice. PKCγ-KO mice showed no obvious changes in step widths; however, the strides were significantly shorter than those of age-matched WT mice (Fig. 1 *E*–*G*). The shorter step strides were significantly restored to WT levels by the rescue of PKCγ in PKCγ-KO mouse PCs (Fig. 1*F*).

### Emergence of Motor Impairment by the Removal of PKCγ from Mature *Prkcg^fl/fl^* Mouse PCs.

**Fig. 2.**
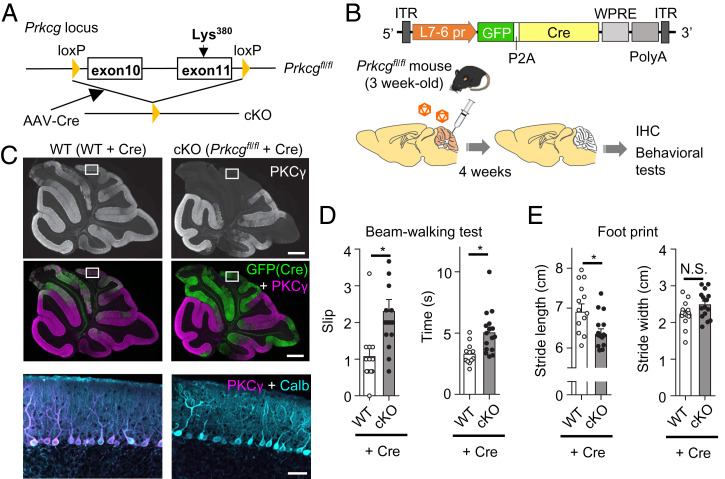
Motor coordination was impaired in adult PC-specific PKCγ-deficient (cKO) mice. (*A*) Schema of *Prkcg* gene floxed (*Prkcg^fl/fl^*) and knockout (cKO) alleles. Exons 10 and 11 of the *Prkcg* gene encoding PKCγ are loxP-flanked. Exon 11 contains a genetic code corresponding to Lys380 critical for ATP binding. Exons 10 and 11 are deleted by AAV vector–mediated expression of Cre recombinase. (*B*) Diagram showing the generation of PC-specific PKCγ KO mice. Three-week-old *Prkcg^fl/fl^* and their littermate WT mice received a cerebellar injection of AAV9 vectors expressing GFP-P2A-Cre by a PC-specific L7-6 promoter at 1.0 × 10^9^ vg/mouse. The cerebella of the treated mice were examined by immunohistochemistry and behavioral tests 4 wk after viral injection. (*C*) Immunohistochemistry of the cerebellar sections from the WT and cKO mice. The sections were stained with antibodies for GFP, PKCγ, and calbindin. *Upper* black-and-white photos show PKCγ immunoreactivity of the whole cerebellar sections. *Middle* panels were same sections as *Upper* ones, but they are presented as green channel (GFP) together with magenta channel (PKCγ). *Lower* photos were magnification of square regions in *Upper* and *Middle* panels, showing immunoreactivity for PKCγ (magenta) and calbindin (blue), a PC marker. Note: A complete loss of PKCγ immunoreactivity selectively in regions expressing GFP (Cre) in cKO mouse PCs. (Scale bars in *Right Upper* and *Lower* panels, 500 and 50 μm, respectively.) (*D*) WT and cKO mice were subjected to the beam-walking test. Graphs show the number of slips and the time spent walking, respectively (WT 12 mice, cKO 16 mice; *P < 0.05 by Welch’s *t* test). (*E*) WT and cKO mice were also subjected to the footprint test. Stride lengths (*Left*) and stride widths (*Right*) are shown in the graphs, respectively (WT 14 mice, cKO 16 mice; *P < 0.05 by Welch’s *t* test). IHC, immunohistochemistry; loxP, locus of crossover in P1; ITR, inverted terminal repeat; N.S., not significant; PolyA, polyadenylation signal sequence; L7-4 pr, L7-4 promoter with minimal cytomegalovirus sequence; WPRE, woodchuck hepatitis virus posttranscriptional regulatory element.

The behavioral improvement of PKCγ-KO mice by AAV vector–mediated re-expression of PKCγ in PCs may be due to the nonphysiological overexpression of PKCγ. To further validate the critical role of PKCγ in motor coordination, we generated PKCγ-flox mice that lost PKCγ expression in a Cre recombinase–dependent manner. These flox mice (*Prkcg^fl/fl^*) possess *loxP* sites flanking exons 10 to 11 of the PKCγ-coding *Prkcg* gene (Fig. 2*A*). The *Prkcg^fl/fl^* mice grew normally and exhibited no motor defects. At 3 wk of age, the *Prkcg^fl/fl^* mice and their WT littermates received a cerebellar injection of AAV9 vectors expressing Cre and GFP under the control of a PC-specific L7-6 promoter (16) (Fig. 2*B*). Immunohistochemistry 4 wk after the viral injection showed the loss of PKCγ in GFP-labeled (Cre-expressing) *Prkcg^fl/fl^* mouse PCs, in contrast with the robust PKCγ immunoreactivity in GFP-labeled (Cre-expressing) WT mouse PCs (Fig. 2*C*). The AAV vector–treated *Prkcg^fl/fl^* mice that lacked PKCγ specifically in their PCs after maturation were referred to as conditional KO (cKO) mice. Beam-walking and footprint tests showed significantly poorer performance in cKO mice than in WT mice treated with AAV vectors expressing Cre (Fig. 2 *D* and *E* and Movie S2). These results confirmed that PKCγ expressed in mature mouse PCs plays a critical role in motor coordination. Slip frequency appeared to be different between naïve WT mice (almost none in Fig. 1*D*) and AAV-injected WT mice (∼1 in Fig. 2*D*). This may be due to physical damage by viral injection and the toxicity of Cre recombinase expression (25). The milder motor defect in cKO mice compared to systemic PKCγ-KO mice is explained by the physically limited spread of AAV vectors and, thus, insufficient Cre-mediated recombination throughout the cerebellum (Fig. 2*C*).

### CF Innervation Profile of PCs Remained Unaffected by Rescue or Deletion of PKCγ.

Next, we examined the consequences of PKCγ rescue on PKCγ-deficient PCs or the deletion of PKCγ from the *Prkcg^fl/fl^* mouse PCs using the whole-cell patch-clamp technique, using cerebellar slices from 7- to 10-wk-old mice (4 to 7 wk postviral injection). We found no statistically significant differences in the PCs between WT mice and KO mice or between Cre-expressing WT mice and Cre-expressing cKO mice in terms of membrane capacitance and ratios of paired-pulse facilitation from the parallel fiber (PF) to the PC synapses, as well as the paired-pulse depression from the CF to the PC synapses (*SI Appendix*, Fig. S1).

**Fig. 3.**
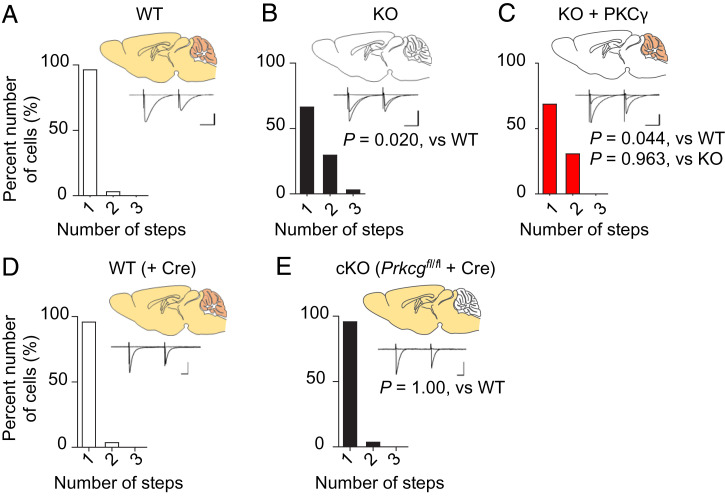
Re-expression or removal of PKCγ did not influence the CF innervation profile of PCs. CF-EPSCs were recorded from whole-cell–clamped PCs by increasing the stimulus intensity. PC frequency histograms in terms of the number of discrete CF-EPSC steps are shown as a graph. (*A*) WT mice. (*B*) PKCγ-KO mice. (*C*) PKCγ-KO mice that received a cerebellar injection of AAV vectors expressing PKCγ under the control of the PC-specific L7 promoter. (*D* and *E*) WT (*D*) and *Prkcg^fl/fl^* (*E*) mice that received a cerebellar injection of AAV vectors expressing Cre under the control of the PC-specific L7 promoter. The horizontal axis shows the number of CF-EPSC steps. Statistical difference was determined by Kruskal–Wallis test. Schema of brain sections showing the presence (bright yellow orange) or absence (blank) of PKCγ. Representative CF-EPSC traces are shown in the graphs. (Scale bars, 20 ms and 400 pA.)

We subsequently tested the CF innervation profiles of PCs. As previously reported (13), approximately one-third of the PKCγ-KO mouse PCs (10 out of 30 cells from four mice) were persistently innervated by multiple CFs (Fig. 3*B*), whereas almost all WT mouse PCs (29 out of 30 cells from four mice) and Cre-expressing WT mouse PCs (25 out of 26 cells from five mice) were innervated by a single CF (Fig. 3 *A* and *D*). Notably, the re-expression of PKCγ in mature PKCγ-KO mouse PCs failed to prune the supernumerary CFs from PCs; a similar proportion of PCs remained innervated by multiple CFs even 4 to 7 wk after the viral injection (9 out of 29 cells from three mice) (Fig. 3*C*). Similarly, the cKO mouse PCs remained innervated by a single CF at 4 to 7 wk after the viral injection (25 out of 26 cells from six mice) (Fig. 3*E*), instead of demonstrating motor impairment (Fig. 2 *D* and *E*). These results suggest that a mechanism distinct from multiple CF innervation of PCs is likely involved in motor defects in PKCγ-deficient mice.

In addition to excitatory synaptic inputs, we assessed the miniature inhibitory postsynaptic currents (mIPSCs) recorded in the PCs. Again, there were no significant differences between the amplitudes and frequencies of picrotoxin-sensitive mIPSCs in WT and PKCγ-KO mice (*SI Appendix*, Fig. S2).

### Significantly Lower CF-Evoked Excitatory Postsynaptic Current Amplitudes in PCs from PKCγ-KO Mice.

**Fig. 4.**
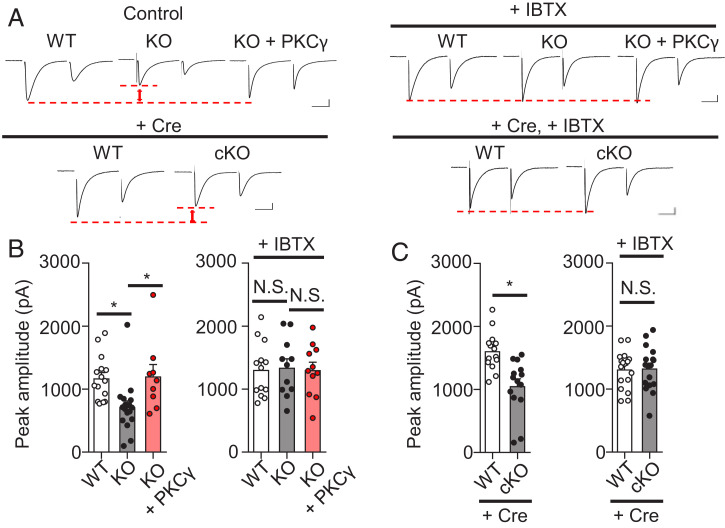
BK channel–dependent lower CF-EPSC amplitudes in PKCγ-KO mouse PCs. CF-EPSCs were elicited in the PCs from WT, PKCγ-KO, and rescue (PKCγ-KO) mice at −10 mV. Similarly, CF-EPSCs were recorded in PCs from WT and cKO (*Prkcg^fl/fl^*) mice that virally expressed Cre in PCs. (*A*) Significantly lower CF-EPSC amplitudes in PKCγ-deficient PCs than in WT mouse PCs. The lower CF-EPSC amplitudes in the PKCγ-KO mouse PCs were restored by PKCγ rescue. (*B* and *C*) Normalization of CF-EPSC size in PKCγ-KO mouse PCs (*B*) and cKO mouse PCs (*C*) by the addition of 100 nM iberiotoxin (IBTX), a BK channel blocker, to the extracellular medium. Stimulation artifacts were removed from the traces. (Scale bars, 20 ms and 200 pA.) Statistical significance was determined by Welch’s *t* test or by Bonferroni post hoc test following one-way ANOVA. N.S., not significant.

As demonstrated above, there was no difference between the electrophysiological properties of WT and PKCγ-KO mice, except for the number of CFs innervating one PC. However, after careful examination, we found that the largest amplitudes of CF-evoked excitatory postsynaptic currents (CF-EPSCs), which were elicited in the KO mouse PCs by applying maximal stimulation to activate all the CFs innervating the recording PC, were significantly reduced to ∼70% of those elicited in the WT mouse PCs (WT: 1,173 ± 90.8 pA, *n* = 15 from five mice, KO: 715.4 ± 99.3 pA, *n* = 17 from five mice) (*P* = 0.0053) (Fig. 4 *A* and *B*). Hereafter, our examination focused on the largest amplitudes of CF-EPSCs. Notably, the lower CF-EPSC amplitude elicited in the KO mouse PCs was restored by the re-expression of PKCγ (1,204 ± 186.8 pA, *n* = 9 from three mice, *P* = 0.026) (Fig. 4 *A* and *B*). A similar decrease in the CF-EPSC amplitude was observed in the cKO mouse PCs (WT: 1,610 ± 82.2 pA, *n* = 14 from four mice, KO: 1,055 ± 114.2 pA, *n* = 14 from four mice, *P* = 0.0006) (Fig. 4 *A* and *C*).

We sought a mechanism that caused a reduction in the CF-EPSCs. Although several reasons could have accounted for the lower EPSC amplitude, a major factor may have been a change in the properties of the postsynaptic α-amino-3-hydroxy-5-methyl-4-isoxazolepropionic acid–type glutamate receptors (AMPARs), such as the single-channel kinetics or the number of surface receptors. In our experiment, the CF-EPSCs were recorded at −10 mV to reduce the current size and avoid the unclamped condition. Given that the AMPAR current–voltage relationship was not altered in the PKCγ-KO PCs (12), the CF-EPSC amplitude should be consistently lower in the KO mouse PCs than in the WT mouse PCs irrespective of the holding potential. We verified this by measuring the CF-EPSCs at −70 mV. To reduce the CF-EPSC amplitudes and record the currents under better clamp conditions, recordings were made in the presence of 0.5 µM 2,3-dioxo-6-nitro-1,2,3,4-tetrahydrobenzo[f]quinoxaline-7-sulfonamide, a non-N-methyl-D-aspartic acid (NMDA) receptor antagonist, in the extracellular solution. We found no significant difference in the CF-EPSC amplitude among the three groups (WT: 1,144 ± 102 pA, *n* = 10 from three mice, KO: 1,103 ± 117 pA, *n* = 7 from three mice, KO + PKCγ: 1,276 ± 103 pA, *n* = 10 from three mice) (*P* = 0.503) (*SI Appendix*, Fig. S3*A*). These results suggest that the reduction of CF-EPSC amplitudes in the KO mouse PCs was not because of a change in the AMPAR properties; rather, it was caused by some factor(s) that emerged with the depolarized membrane voltage.

### CF-EPSC Amplitudes Were Attenuated by Large-Conductance Calcium-Activated Potassium Channels.

We did not expect smaller CF-EPSCs in the PCs from the PKCγ-KO mice, because the original study by Kano et al. (13) showed no significant difference in the CF-EPSC amplitudes between WT mice and KO mice. In our study, we used ethylene glycol-bis(β-aminoethyl ether)-N,N,N′,N′-tetraacetic acid (EGTA) as an intracellular Ca^2+^ buffer, while a previous study used 1,2-bis-(*o*-aminophenoxy)-ethane-N,N,N′,N′-tetraacetic acid (BAPTA) (13). Thus, we assumed that the difference in the intracellular Ca^2+^ buffer might explain the reduction in the CF-EPSC amplitude in PKCγ-KO mouse PCs. To prove this, we used 10 mM BAPTA as a Ca^2+^ buffer and recorded CF-EPSCs at −10 mV. Under these conditions, we found no difference in the CF-EPSC amplitudes between WT mice and KO mice (WT: 1,253 ± 87.8 pA, *n* = 15 from four mice, KO: 1,115 ± 86.3 pA, *n* = 12 from four mice) (*P* = 0.565) (*SI Appendix*, Fig. S3*B*). Since BAPTA chelates internal Ca^2+^ transients much faster than EGTA (26, 27), some voltage-dependent and fast kinetic Ca^2+^ events in PCs likely reduced the CF-EPSC amplitudes in the PKCγ-KO mouse PCs. When depolarized, a large Ca^2+^ influx was induced in PCs through the activation of VGCCs (28–30). Therefore, we speculated that some Ca^2+^-dependent channels associated with VGCC activation play a key role in the reduction of CF-EPSC amplitudes in PKCγ-KO mouse PCs.

Calcium-activated potassium channels, which require Ca^2+^ influx through VGCCs for activation, are expressed in PCs (31, 32). Thus, we tested whether the reduction of CF-EPSC amplitudes in PKCγ-KO mouse PCs was associated with the activation of K^+^ channels by recording CF-EPSCs in the presence of 500 µM tetraethylammonium (TEA), a nonselective K^+^ channel blocker. Under these conditions, we could not detect significant differences in the CF-EPSC amplitude between WT mice and KO mice (WT: 1,016 ± 62.6 pA, *n* = 14 from four mice, KO: 1,024 ± 146.3 pA from four mice, *n* = 12) (*P* = 0.793) (*SI Appendix*, Fig. S3*C*), suggesting the involvement of K^+^ channel activity in the reduction of the CF-EPSC amplitudes in PKCγ-KO mouse PCs.

PCs express BK channels, which are activated by Ca^2+^ influx through P/Q-type VGCCs (33). In the dorsal cochlear nucleus, BK channels constitute nanodomains with ryanodine receptors and VGCCs (34). Since Ca^2+^ buffering by a chelator is limited by the distance from the Ca^2+^ source (18, 35), BAPTA, but not EGTA, was shown to block BK channel activation (34). In PCs, BK channels are located 40 nm from P/Q-type VGCCs on average (19). Thus, we can reasonably suppose that BK channel activation is blocked by BAPTA, thereby masking the attenuating effect of BK channels on CF-EPSC responses in PKCγ-KO PCs (13). We validated this hypothesis by recording CF-EPSCs in the presence of 100 nM iberiotoxin, a BK channel–specific blocker. Under these conditions, we did not observe significant differences between the CF-EPSC amplitudes of the WT and PKCγ-KO mice (WT: 1,306 ± 120 pA, *n* = 13 from three mice, KO: 1,342 ± 138 pA, *n* = 11 from three mice, *P* = 0.971) and between the Cre-expressing WT and cKO mice (Cre-expressing WT: 1,314 ± 113 pA, *n* = 18 from three mice, cKO: 1,327 ± 112 pA, *n* = 17 from three mice, *P* = 0.905) (Fig. 4 *A–C*). These results suggest that the BK channel function is suppressed by PKCγ, thereby increasing the size of the CF-EPSCs.

### Significantly Larger BK Channel Currents in PKCγ-KO PCs than in WT PCs.

**Fig. 5.**
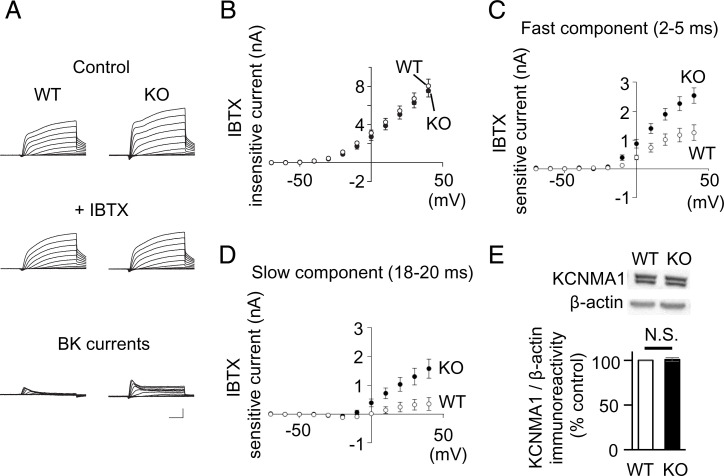
Significantly larger BK currents were seen in PKCγ-KO mouse PCs. (*A*) Representative raw potassium currents evoked in the WT and PKCγ-KO mouse PCs by 20-ms steps from −70 mV to +40 mV in 10-mV increments in the extracellular solution without (control, *Top*) or with 100 nM iberiotoxin (+IBTX, *Middle*). The net BK currents (*Bottom*) were calculated by subtracting the traces elicited with iberiotoxin (*Middle*) from the control traces (*Top*). (Scale bars, 5 ms and 2 nA.) (*B*) Graph of values of iberiotoxin-insensitive currents elicited in the WT and PKCγ-KO mouse PCs at various potentials. (*C* and *D*) BK current sizes at peaks of fast components (2 to 5 ms after the current onset) and stable current of slow components (18 to 20 ms after the current onset) plotted in the graphs. Both fast and slow components were significantly larger in PKCγ-KO mouse PCs than in WT mouse PCs (*P* = 0.0039 and *P* = 0.016, respectively, by repeated-measures ANOVA). (*E*) No significant difference in the amount of protein in the BK channels in the WT and PKCγ-KO mouse cerebella. The cerebellar hemispheres were subjected to Western blotting. Quantitative analysis of the band intensities immunoreactive to pore-forming BK channel α-subunits (KCNMA1). Each band intensity was normalized to that of β-actin. N.S., no significant difference was determined using Welch’s *t* test (*P* = 0.732).

PKC phosphorylation of BK channels is known to inhibit open probability in transfected HEK293 cells (36). Likewise, PKCγ may regulate the BK channel function in PCs. To verify this, we recorded BK channel–mediated currents from WT and PKCγ-deficient PCs. To prevent Ca^2+^ spike generation in unclamped distal dendrites in mature and well-differentiated PCs, BK currents were recorded from the PCs of P10 to P14 mice. The currents were evoked in extracellular solutions containing 5 mM 4-aminopyridine and 1 µM tetrodotoxin to block Ca^2+^-independent voltage-gated K^+^ currents and voltage-gated sodium channel currents, respectively, by 20-ms step depolarization pulses in 10-mV increments from a holding potential of −70 mV. The voltage steps were repeated in the presence of 100 nM iberiotoxin. The remaining currents, namely the iberiotoxin-insensitive currents, were comparable in the WT (*n* = 9 from three mice) and PKCγ-KO (*n* = 9 from three mice) mouse PCs (*P* = 0.543) (Fig. 5 *A* and *B*). The BK currents (Fig. 5*A*, *Bottom* traces) were obtained by subtracting the iberiotoxin-insensitive currents from the original (control) currents (before application of iberiotoxin). The BK currents, which were elicited at depolarized membrane voltages over −20 mV, were constituted by an initial peak (named as a fast component) and a subsequent stable tail current (named as a slow component). Both fast and slow components were consistently larger in PKCγ-KO mouse PCs than in WT mouse PCs (*P* = 0.0039 and *P* = 0.016, respectively) (Fig. 5 *C* and *D*). Incubation of cerebellar slices with 5 µM chelerythrine, a membrane-permeable PKC inhibitor, increased the BK currents in WT mouse PCs to a size comparable to that in PKCγ-KO mouse PCs (*P* = 0.6407) (*SI Appendix*, Fig. S4), suggesting suppression of BK currents by PKCγ.

Since PKCγ might regulate the expression levels of BK channels in PCs, we compared the protein levels of BK channels between WT mouse cerebella and those of PKCγ-KO mice by Western blotting. To minimize the contamination of deep cerebellar nuclei that also express BK channels (37, 38), we used the cerebellar hemisphere for the analysis. Western blotting revealed that there was no significant difference in protein expression levels of the pore-forming BK channel α-subunit (KCNMA1) between WT and PKCγ-KO mouse cerebella (both from three mice) (*P* = 0.732) (Fig. 5*E*), suggesting that PKCγ regulates function, but not the expression levels of the BK channel in PCs.

### PKCγ Increases Membrane Resistance and Prevents Decay of CF Signal in PCs.

**Fig. 6.**
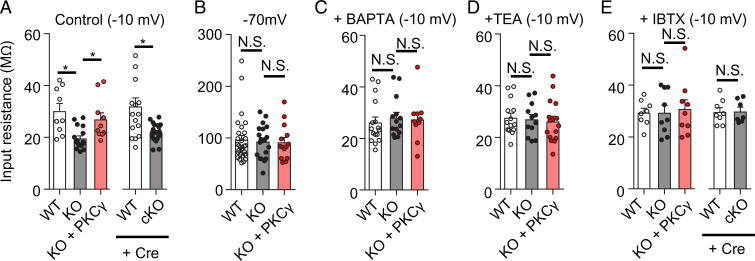
PKCγ increases the membrane resistance of PCs through BK channels. (*A*) Control membrane resistance recorded from the PCs, as depicted at −10 mV using a standard extracellular medium and an internal solution containing EGTA. (*B*) Membrane resistance was recorded at −70 mV. (*C*) Membrane resistance recorded at −10 mV using an internal solution containing BAPTA instead of EGTA. (*D*) Membrane resistance recorded at −10 mV in the presence of TEA in the extracellular medium. (*E*) Membrane resistance recorded at −10 mV in the presence of iberiotoxin (IBTX) in the extracellular medium. Statistical significance was determined by Welch’s *t* test or by Bonferroni post hoc test following one-way ANOVA. N.S., not significant.

BK channels are richly expressed along the proximal dendrites to the soma of PCs, where these channels form clusters in the vicinity of VGCCs at a distance of only a nanometer (19, 20). CF input depolarizes a PC and induces Ca^2+^ influx through the opening of VGCCs, and subsequent activation of BK channels is thought to reduce membrane resistance, resulting in electrical shunting along the proximal dendrites to soma in PCs. Therefore, larger BK currents in PKCγ-KO mouse PCs most likely reduce membrane resistance and attenuate CF signal transduction through the dendrites. To validate this hypothesis, we compared the membrane resistance of WT, PKCγ-KO, and PKCγ-rescued KO mouse PCs. As expected, at −10 mV (control condition), membrane resistance was significantly lower in PKCγ-KO mouse PCs (19.6 ± 1.0 MΩ, *n* = 17 from four mice) than in WT mouse PCs (30.2 ± 3.0 MΩ, *n* = 9 from three mice, *P* = 0.0017), which was reversed by re-expression of PKCγ in KO mouse PCs (27.0 ± 2.5 MΩ, *n* = 10 from three mice, vs. KO; *P* = 0.0089) (Fig. 6*A*). A decrease in membrane resistance was also observed in the cKO mouse PCs (WT + Cre; 32.0 ± 3.3 MΩ, *n* = 16 from four mice, cKO; 21.9 ± 0.7 MΩ, *n* = 21 from four mice, *P* = 0.008) (Fig. 6*A*).

A decrease in the input resistance of the PKCγ-deficient PCs was not observed at −70 mV (Fig. 6*B*). Even at −10 mV, the lower input resistance in the PKCγ-deficient PCs was not detected by the addition of BAPTA to the internal solution, or in the presence of TEA or iberiotoxin in the external solution (Fig. 6 *C*–*E*).

Because electrical shunting accelerates signal kinetics, we compared the decay time constants of the CF-EPSCs in the WT, PKCγ-deficient, and PKCγ-rescued PCs. Decay of CF-EPSC at −10 mV was faster in PKCγ-KO mouse PCs (7.92 ± 0.46 ms, *n* = 18 from five mice) than in WT mouse PCs (9.82 ± 0.60 ms, *n* = 15 from five mice) (*P* = 0.042) (*SI Appendix*, Fig. S5*A*). Faster decay was observed in cKO mouse PCs (WT: 8.51 ± 0.60 ms, *n* = 12 from four mice, cKO: 6.93 ± 0.32 ms, *n* = 12 from four mice) (*P* = 0.020) (*SI Appendix*, Fig. S5*A*). However, this was not restored by reexpression of PKCγ to PKCγ-KO mouse PCs (8.68 ± 0.07 ms, *n* = 9 from five mice) (vs. KO: *P* = 0.999) (*SI Appendix*, Fig. S5*A*). These results suggest that some developmental factors underlie the faster decay of CF-EPSCs in PKCγ-KO mouse PCs.

The faster decay of the CF-EPSCs in the PKCγ-KO mouse PCs at −10 mV was not detected by shifting the membrane voltage to −70 mV or by addition of BAPTA to the internal solution; however, it remained even in the presence of TEA or iberiotoxin in the external solution (*SI Appendix*, Fig. S5 *B*–*E*). Thus, some voltage- and calcium-dependent components other than the K^+^ channels most likely regulate the decay time constant of CF-EPSCs in PKCγ-deficient PCs.

### No Significant Difference in CF-Evoked Ca^2+^ Signaling Was Observed between WT and PKCγ-KO Mouse PCs.

CF synaptic inputs to PCs evoke Ca^2+^ elevation in PC dendrites (39). If the degree of Ca^2+^ elevation at the PC dendrites in response to CF inputs differs between WT mice and PKCγ-KO mice, it affects the extent of BK channel activation. To examine whether PKCγ deficiency affects CF-evoked Ca^2+^ signals in PCs, we performed confocal live Ca^2+^ imaging experiments (*SI Appendix*, Fig. S6). After maximizing the CF responses, the CF-evoked Ca^2+^ signals were recorded from the PCs in the current-clamp mode, where physiological Ca^2+^ signals were accordingly observed. Although the CF-evoked Ca^2+^ signals were quite variable between the PCs (*SI Appendix*, Fig. S6*B*), as reported in a previous study (40), there was no significant difference between the CF-evoked Ca^2+^ signals in the WT (*n* = 8 from four mice) and PKCγ-KO (*n* = 10 from four mice) mice (*SI Appendix*, Fig. S6*B*; Ca^2+^ peak [ΔF/F_b_], WT 0.57 ± 0.05, PKCγ-KO 0.52 ± 0.07, *P* = 0.56; Ca^2+^ integral [ΔF/F_b_·ms], WT 141 ± 16, PKCγ-KO 137 ± 21, *P* = 0.89; Ca^2+^ decay [ms], WT 189 ± 28, PKCγ-KO 191 ± 18, *P* = 0.94). This indicates that PKCγ-KO mice have no abnormalities in CF-induced Ca^2+^ signaling in PCs.

### Kinase Activity of PKCγ Is Indispensable to the Rescue of Aberrant Phenotypes in PKCγ-KO Mice.

To test whether the kinase activity of PKCγ plays an essential role in the rescue of aberrant phenotypes in PKCγ-KO mice, we used kinase-negative PKCγ (PKCγ-KN), which has a mutation at the adenosine triphosphate (ATP)-binding site (K380M) to avoid substrate phosphorylation (41). AAV9 vectors expressing GFP-P2A-PKCγ-KN were injected into the cerebellum of PKCγ-KO mice (*SI Appendix*, Fig. S7 *A* and *B*). Four weeks after viral injection, PKCγ-KN expression was confirmed by immunohistochemistry (*SI Appendix*, Fig. S7*C*). Beam-walking and footprint tests showed no significant difference between naïve PKCγ-KO mice and those expressing PKCγ-KN (*SI Appendix*, Fig. S7*D* and Movie S3). Moreover, we found no significant differences between the electrophysiological properties, such as the amplitude and decay time constant of the CF-EPSCs and the membrane resistance between the naïve PKCγ-KO mice and those expressing PKCγ-KN (*SI Appendix*, Fig. S7 *E*–*G*). These results suggest that PKCγ rescues aberrant phenotypes in a kinase activity–dependent manner.

### Altered Complex Spike Shapes with Significantly Fewer Spikelets Were Observed in the PKCγ-Deficient PCs.

The CF synaptic input generates a complex spike composed of an initial large action potential with subsequent smaller spikelets (Na^+^ spikes) that are produced at the axon initial segment and are superimposed on sustained depolarization (42). As described above, CF inputs caused similar degrees of Ca^2+^ transients in the PKCγ-deficient mouse PCs as in the WT mouse PCs; however, it seems likely that, because of the enhanced BK channel function, electrical signals propagating along dendrites decay faster in PKCγ-deficient mouse PCs than in WT mouse PCs. To test this possibility, we recorded CF-evoked potentials at the PC soma and compared the amplitudes between cKO and WT mice. The intracellular solution contained 1 mM QX-314, a membrane-impermeable voltage-gated Na^+^ channel blocker, to selectively suppress Na^+^ spikes in PCs. Before starting the experiment, the membrane potential of the recording PC was adjusted to −70 mV by current injection, followed by electrical stimulation of the granule cell layer. CF-evoked depolarization was successfully recorded in the soma of WT and cKO mouse PCs. The amplitudes from the baseline (−70 mV) to the peak (ΔmV) were significantly lower in cKO mouse PCs than in WT mouse PCs for ∼10 mV (WT: 58.5 ± 1.8 mV, n = 8 from three mice, cKO: 48.7 ± 3.6 mV, n = 10 from three mice, P = 0.030) (*SI Appendix*, Fig. S8).

**Fig. 7.**
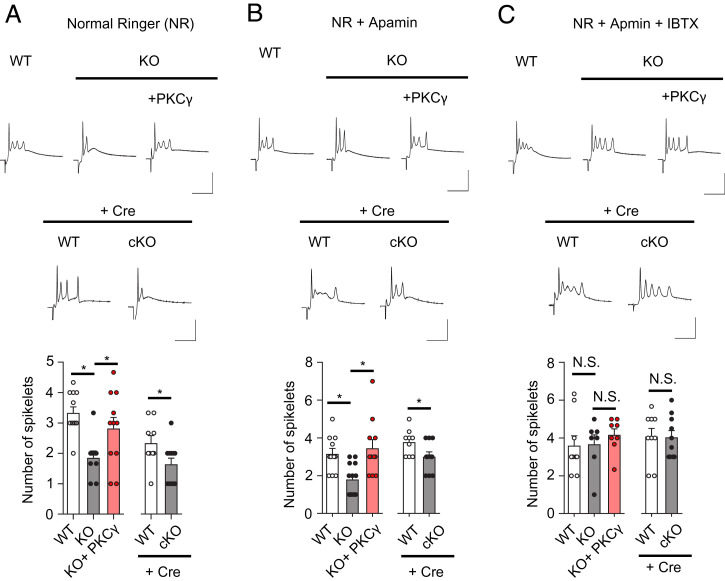
Significant decreases in spike number on complex spikes. (*A*) The number of spikes superimposed on the sustained depolarization, except for the first one, was counted from the PCs. A significant decrease in the spikes was observed in the PKCγ-deficient PCs (KO and cKO), which was restored by the AAV-mediated reexpression of PKCγ (KO + PKCγ). (*B*) The addition of apamin, an SK channel blocker, to the extracellular medium did not influence the number of spikes. (*C*) Significant rescue of the number of spikes in the PKCγ-deficient PCs (KO and cKO) by the addition of iberiotoxin (IBTX), a BK channel blocker, to the extracellular medium. Representative CF-EPSP traces are shown above each graph. (Scale bars, 10 ms and 50 mV.) Statistical significance was determined by Welch’s *t* test or Bonferroni post hoc test following one-way ANOVA. N.S., not significant.

Thus, we supposed that the CF signal attenuated during propagation to the axon initial segment, resulting in a lower efficacy of Na^+^ spike generation, and most likely it altered complex spike shapes in the PKCγ-KO mouse PCs. To prove this, we assessed the number of spikelets on each complex spike. As shown in Fig. 7*A*, the average number of spikelets per complex spike was significantly lower in the PKCγ-KO mouse PCs (1.85 ± 0.23, *n* = 9 from three mice) than in the WT PCs (3.33 ± 0.19, *n* = 12 from three mice, *P* = 0.0015). This was rescued by the reexpression of PKCγ in KO mouse PCs (KO + PKCγ; 2.82 ± 0.36, *n* = 11 from three mice, *P* = 0.046, vs. PKCγ-KO). Fewer spikelets per complex spike were observed in the cKO mouse PCs (WT: 2.33 ± 0.25, *n* = 9 from three mice, cKO: 1.64 ± 0.20, *n* = 11 from three mice, *P* = 0.045) (Fig. 7*A*).

In addition to BK channels, PC dendrites express small conductance Ca^2+^-activated potassium (SK) channels, which have been shown to possess a consensus sequence for PKC phosphorylation (43). Thus, the altered CS waveform observed in PKCγ-KO mouse PCs may be caused by modulation of SK channel activity. To examine this possibility, we first applied 10 µM apamin, a selective SK channel blocker. The fewer spikelets per complex spike in the PKCγ-KO mouse PCs and cKO mouse PCs were not influenced by perfusing the slices with apamin; however, they were normalized by blocking the BK channels with iberiotoxin (50 nM) (Fig. 7 *B* and *C*).

### PKCγ and BK Channels Regulate CF–Long-Term Depression Expression.

Long-term depression (LTD) of synaptic transmission can be induced in CF-PC and PF-PC synapses (44), both of which are not induced in the presence of a PKC inhibitor (44, 45). Previous studies revealed that PKCα, and not PKCγ, plays a critical role in the expression of LTD at PF-PC synapses (PF-LTD) (46), whereas a PKC isoform that is critical for CF-LTD expression has not yet been clarified. The involvement of PKCα in PF-LTD expression is reasonable on account of its close localization at the postsynaptic sites of PF-PC synapses (47, 48). Likewise, PKCγ, which is located along the dendritic shafts (8), likely plays a critical role in CF-LTD expression. To test this hypothesis, we examined whether CF-LTD could be induced in PKCγ-KO mouse PCs. In WT mouse PCs, tetanic stimulation of CF at 5 Hz for 30 s reliably induced CF-LTD, and the CF-EPSC amplitude was ∼80% of the original level, as previously demonstrated (44). In contrast, a similar tetanic stimulation failed to induce CF-LTD, and CF-EPSC amplitude remained almost unchanged 30 min after tetanic stimulation in PKCγ-KO mouse PCs (*SI Appendix*, Fig. S9). Notably, CF-LTD expression in WT mouse PCs was completely blocked in the presence of iberiotoxin, a BK channel blocker (*SI Appendix*, Fig. S9). These results suggest the critical involvement of PKCγ and BK channels in CF-LTD expression.

## Discussion

In this study, using systemic and conditional PKCγ-deficient mice, we sought to clarify the physiological significance of PKCγ, a major isotype of cPKC, which is abundantly expressed throughout the dendrites and soma of PCs (8, 10). We found that PKCγ is a critical regulator that negatively modulates BK currents in PC dendrites by modulation of dendritic signal transduction. PKCγ-deficient mice and BK channel–KO (BK^−/−^) mice served as the maximum BK current model and loss of the BK current model, respectively (*SI Appendix*, Fig. S10).

BK channel activity is affected by intracellular Ca^2+^ concentration: elevation of intracellular Ca^2+^ concentration shifts the voltage sensitivity to more hyperpolarized potentials (49). In PCs, the main Ca^2+^ source for BK channel activation is P/Q-type VGCCs (19), and enhanced VGCC activation may be responsible for the increase in the BK currents in PKCγ-KO mouse PCs (Fig. 5). However, consistent with a previous study showing similar kinetics and amplitude of VGCC currents in WT and PKCγ-KO mouse PCs (12), our Ca^2+^ imaging experiment showed no significant difference between WT and PKCγ-KO mouse PCs (*SI Appendix*, Fig. S6), indicating that Ca^2+^ supply to BK channels is normal in PKCγ-KO mouse PCs. In addition, the voltage sensitivity of BK channels was almost identical in WT and PKCγ-KO mouse PCs (Fig. 5). Thus, we concluded that the absence of PKCγ resulted in enhanced BK channel activity in PCs—that is, activation of PKCγ suppresses BK channel activity (*SI Appendix*, Fig. S10), analogous to the medial vestibular nucleus neurons, in which activation of PKC decreased the BK channel open probability (50).

BK channels in PCs are clustered and distributed along the proximal dendrites to the soma in close vicinity to the VGCCs (19, 20). Previous studies, together with our data, suggest that BK channels exert at least three influences on PCs. First, BK channels contribute to hyperpolarization (AHP) following the action potential (38). Electrophysiological analysis of PCs from ataxic BK^−/−^ mice showed reduced amplitude of the AHP and substantial reduction of PC firing, the latter of which was suggested to be mediated by depolarization-induced Na^+^ channel inactivation (38). Second, BK channels are thought to regulate dendritic Ca^2+^ transients upon CF synaptic input, since PC-specific BK^−/−^ mice showed a marked increase in CF-evoked Ca^2+^ transients in PCs, compared with WT mice (51). However, in our study, CF-evoked Ca^2+^ signaling was almost comparable between WT and PKCγ-KO mouse PCs (*SI Appendix*, Fig. S6). An increase in CF-evoked Ca^2+^ transients in PC-specific BK^−/−^ mouse PCs could be a homeostatic compensation since the BK^−/−^ mouse PCs show a marked decrease in CF inputs (51). Alternatively, defects in PKCγ may not significantly affect local Ca^2+^ transients in PCs. Third, the BK channels modulate the membrane resistance, as presented in Fig. 6. BK channel activation causes a shunting effect, which decays the electrical signal strength during its conduction through the dendrites.

In addition to BK channels, PC dendrites express SK channels, which are distributed up to the distal dendritic branchlets (52, 53). The PF burst input enhances the spike count in the PC, a phenomenon called intrinsic plasticity. Intrinsic plasticity, which enhances PC excitability, is mediated by the downregulation of SK channels (52). Although the BK channel is not involved in the expression of intrinsic plasticity, BK channel inhibition by iberiotoxin causes a similar enhancement of PC excitability (52), suggesting that BK channels can also modulate PC excitability. Thus, modulation of BK currents by PKCγ could regulate PF signal conduction. Moreover, it may be possible that PKCγ modulates SK channel function, as SK channels are shown to possess a consensus sequence for PKC phosphorylation (43).

Recent studies have proposed that SK channel–dependent intrinsic plasticity regulates signal weight from individual dendritic branches (54–56). Considering the difference in spatial expression profiles between the two Ca^2+^-activated K^+^ channels, BK channels are assumed to act at proximal dendrites as the last standing filter of dendritic signal conduction to the soma, while SK channels might be involved in the modulation of signal conduction at each dendritic branch.

Previously, CF-LTD was shown to be induced following brief tetanic stimulation (5 Hz, 30 s) to CF, whose expression was blocked by the addition of BAPTA in the pipette (intracellular) solution or in the presence of chelerythrine, a membrane-permeable PKC inhibitor (44), suggesting critical roles of Ca^2+^ and PKC in CF-LTD expression. Consistently, the absence of PKCγ in PKCγ-KO mouse PCs or inhibition of BK channels with iberiotoxin in WT mouse PCs failed to induce CF-LTD (*SI Appendix*, Fig. S9). As described in *SI Appendix*, Fig. S10, in WT mouse PCs, we assumed that tetanic stimulation dephosphorylated BK channels by activation of a phosphatase such as protein phosphatase 1 (57), and it eventually enhanced the BK currents. Together with the results that application of a PKC inhibitor increased BK currents in WT mouse PCs (*SI Appendix*, Fig. S4), we surmise that treatment with a PKC inhibitor dephosphorylated BK channels, analogous to those in PKCγ-KO mouse PCs, and thus, failed further dephosphorylation upon tetanic stimulation (44).

After dendritic computation in PCs, PF and CF signals are converted to simple and complex spikes, respectively, at axon initial segments as signal outputs to the deep cerebellar nuclei (DCN) (58). Thus, the dendritic modulation of CF signals by BK channels can affect the complex spike waveform. Indeed, as depicted in *SI Appendix*, Fig. S10, potentiation of BK channel currents by defects in PKCγ in PCs decreased the spikelet number, which was reversed by re-expression of PKCγ (Fig. 7), whereas genetic or pharmacological inhibition of BK channels in PCs increased the spikelet number (51, 59).

Simple spike pausing time following complex spikes becomes shorter when BK channels are pharmacologically blocked (60). Simple spike pausing after complex spikes causes rebound burst activity in the DCN (61). Such hyperactive DCN neurons exert a marked inhibitory action on the target inferior olivary nucleus, and eventually, CF signals from the olivary neurons to PCs are strongly suppressed. It has been suggested that olivo-cerebellar circuits provide timing for motor coordination (62–64). In this context, BK channels can regulate motor coordination through dendritic computation in PCs. Consistent with this notion, PC-specific BK^−/−^ mice showed impaired activity (marked suppression) of olivo-cerebellar circuits, resulting in motor incoordination (51). The roles of BK channels in PCs have been well studied; however, the physiological regulation of BK channel function in PCs remains unclear. Although further studies are necessary, our present results suggest that PKCγ is a critical regulator of the BK channel function in PCs, and thereby contributes to motor coordination.

## Materials and Methods

PKCγ-KO mice (11) were provided by Dr. Masanobu Kano (University of Tokyo, Japan). *Prkcg^fl/fl^* mice were generated using the CRISPR/Cas9 system (65). Mice were maintained on a C57BL/6J genetic background in our breeding colony at the Institute of Experimental Animal Research, Gunma University Graduate School of Medicine. Homozygous PKCγ-KO mice and their WT littermates, or *Prkcg^fl/fl^* mice and their WT littermates, were obtained by crossing fertile heterozygous animals and genotyped by PCR. All procedures related to the care and treatment of animals were performed according to the Japanese Act on the Welfare and Management of Animals. The experimental protocol was approved by the Institutional Committee of Gunma University (No. 20-053 and 21-014). Details of the experimental methods and statistical analyses are included in *SI Appendix*, *Materials and Methods*.

## Supplementary Material

Supplementary File

Supplementary File

Supplementary File

Supplementary File

## Data Availability

All study data are included in the article and/or supporting information.
