# Supplementary material for: Protein kinase Cγ in cerebellar Purkinje cells regulates Ca2+-activated large-conductance K+ channels and motor coordination
Source: Proc Natl Acad Sci U S A. 2022 Feb 10;119(7):e2113336119. doi: 10.1073/pnas.2113336119 (PMC8851492; doi:10.1073/pnas.2113336119)
Supplement: Supplementary File [file pnas.2113336119.sapp.pdf]

SI Materials and Methods

Preparation of adeno-associated virus vectors and cerebellar injection

We used pAAV-L7-4-minCMV-GFP-P2A-PKCγ/PKCγ-KN-woodchuck hepatitis virus post-transcriptional regulatory element (WPRE) or pAAV-L7-6- GFP-P2A-Cre-WPRE for AAV9 vector production. The AAV9 vectors were designed to express PKCγ/PKCγ-KN or Cre together with GFP under the control of the PC-specific L7-4 promoter with the minCMV [1, 2] or L7-6 promoter [3]. P2A, a self-cleaving peptide sequence, was inserted between GFP and PKCγ/PKCγ-KN or Cre, which freed the GFP from the conjugated protein via ribosomal skipping between glycine and proline [4]. The WPRE sequence was inserted following PKCγ/PKCγ-KN or Cre.

Recombinant single-strand AAV9 vectors were produced by transfection of HEK293T cells (Thermo Fisher Scientific, Waltham, MA) with the pAAV expression plasmid pAAV2/9 (kindly provided by Dr. J. Wilson) and a helper plasmid (Stratagene, La Jolla, CA). The genomic titers of the viral vector were determined by real-time quantitative PCR using the Power SYBR Green PCR Master Mix (Thermo Fisher Scientific) with the primers 5-CTGTTGGGCACTGACAATTC-3 and 5-GAAGGGACGTAGCAGAAGGA-3, which targeted the WPRE sequence. The expression plasmid was used as the standard.

Viral vector injection was performed as described previously [5]. The genomic titer was 1.6×10^{11} or 1.6×10^{12} vg/ml for pAAV-L7-4-minCMV-GFP-P2A-PKCγ (or PKCγ-KN)-WPRE and 1.0×10^{11} vg/ml for pAAV-L7-6-GFP-P2A-Cre-WPRE. The syringe was left in place for 2 min following the injection. After closing the scalp, the mice were returned to their standard cages.

Beam-walking test

To evaluate motor performance, the mice were subjected to a beam-walking test. The beam consisted of a 100-cm horizontal steel round bar (11 mm in diameter) placed 50 cm above the floor. After the habituation trials, the mice were placed at the starting point of the bar. The slip number of the fore or hind limb from the bar and the time to cross the 80-cm distance on the bar were measured accordingly. Sessions consisting of three trials per day with a 10-minutes inter-trial interval were conducted for these mice.

Footprint test

Footprints were made using waterproof black ink and white paper. Ink was applied to the hind paws of the mice. The mice were allowed to walk forward in a narrow alley. For each mouse, the stride length and width were measured and accordingly averaged.

Electrophysiological experiments

Electrophysiological experiments were performed using parasagittal cerebellar slices (250 μm in thickness) prepared from P49-69 mice, as described previously [5, 6]. Slices were perfused in an extracellular solution containing 125 mM NaCl, 2.5 mM KCl, 1.25 mM NaH_2PO_4, 26 mM $NaHCO_3$, 2 mM $CaCl_2$, 1 mM $MgCl_2$, and 10 mM glucose, and bubbled continuously with a mixture of 95% O_2 and 5% CO_2 at room temperature. The resistance of the patch pipette was 2–5 MΩ when filled with intracellular solution containing (in mM): 122.5 Cs methanesulfonate, 17.5 CsCl, 8 NaCl, 2 Mg adenosine triphosphate (Mg-ATP), 0.3 Na guanosine triphosphate (Na-GTP), 10 4-(2-hydroxyethyl)-1-piperazineethanesulfonic acid (HEPES), and 0.2 EGTA (adjusted with CsOH). Some experiments used an intracellular solution containing 10

mM BAPTA instead of EGTA. The intracellular solution used to measure the CF-evoked potentials contained an additional 1 mM QX-314. The intracellular solution used for mIPSC recording contained (in mM): 127.5 CsCl, 2 mM $CaCl_2$, 1 mM $MgCl_2$, 2 Mg-ATP, 0.3 Na-GTP, and 10 mM HEPES (pH 7.3, adjusted with CsOH). The intracellular solution used for the BK current and complex spike recording contained (in mM): 122.5 K-gluconate, 17.5 CsCl, 8 NaCl, 10 HEPES, 0.2 EGTA, 2 Mg-ATP, and 0.3 Na-GTP (pH 7.2, 290-300 Osm). Patch-clamp recordings were performed from PCs in lobules Ⅰ - Ⅲ of the cerebellar vermis.

Voltage-clamp recordings

Input resistance and membrane capacitance were estimated from passive currents induced by applying hyperpolarizing pulses (from -70 to -80 mV or -10 to -20 mV, 200 ms duration). PF-EPSCs, CF-EPSCs, and mIPSCs were recorded as described previously [6]. CF-EPSCs were recorded at -70 mV under some conditions. Multiple CF innervation was estimated as described previously [7]. In other experiments recording CF-EPSCs, maximal stimulation was applied to activate all the CFs innervating the individual PCs. CF-LTDs were recorded from P14-17 mouse PCs as described previously [8]. Some BK currents were measured from P10-14 mouse PCs. The PCs were first held at -100 mV for 500 ms, and the currents were then evoked by moving the holding potentials to various potentials. BK currents were calculated by subtracting the traces before and after the application of 100 nM iberiotoxin (4235-s, Peptide Institute Inc., Osaka, Japan). The leak subtraction method was used to record the BK currents to compensate for the leak current. The extracellular solution contained 0.1 mM picrotoxin, except for the solution used to record mIPSCs, which contained 1 μM TTX. The extracellular solution used to record the BK

currents contained 1 μM TTX and 5 mM 4-AP and the solution that was used to record the CF-EPSCs at -70 mV contained 0.5 μM NBQX. In some experiments, the extracellular medium contained 500 μM TEA. Data were discarded when the resistance values changed by >20% of the basal value during the experiment.

Current-clamp recordings

Before starting the experiments, the baseline membrane potentials of the recording PCs were adjusted to approximately -70 mV. CF-mediated depolarization and complex spikes were evoked by the CF stimulation. To evaluate the complex spike waveform, we counted the number of Na^+ spikes superimposed on the sustained depolarization of the complex spike, whose amplitudes were over 5 mV. Some recordings were made in the presence of 10 nM apamin (4257-v, Peptide Institute Inc., Osaka, Japan) and/or 100 nM iberiotoxin.

Immunohistochemistry

The mice were sacrificed 2-4 weeks after viral injection. These deeply anesthetized mice were perfused, and their cerebellar slices were obtained as described previously [5]. The slices were treated with the following primary antibodies: rat monoclonal anti-GFP (1:1,000; Cat. No. 04404-84, Nacalai Tesque, Kyoto, Japan), mouse monoclonal anti-calbindin D-28K (1:500; Cat. No. 300(F), SWANT, *Bellinzona*, Switzerland), and rabbit monoclonal anti-PKCγ (1:1000; Cat. No. AB_2571824; Frontier Institute, Ishikari, Japan) and the following secondary antibodies: Alexa Fluor 488 donkey anti-rat IgG (1:1000; Thermo Fisher Scientific), Alexa Fluor 568 donkey anti-rabbit IgG (1:1000; Thermo Fisher Scientific), and Alexa Fluor 680 donkey anti-mouse IgG

(1:500; Thermo Fisher Scientific). Bright-field and fluorescence images were obtained using a fluorescence microscope (VB-7010 or BZ-X700, Keyence, Osaka, Japan) or a confocal laser-scanning microscope (LSM 800; Carl Zeiss, Oberkochen, Germany).

Confocal Live Calcium imaging

CF-evoked Ca^{2+} signals in the cerebellar PCs were examined by confocal microscopy using an upright microscope (BX51WI, Olympus) equipped with a 40× water immersion objective (LUMPLFLN 40XW, Olympus, Tokyo, Japan), a water-cooled CCD camera (iXon3 DU-897E-CS0-#BV-500, Andor, UK), and a high-speed spinning-disc confocal unit (CSU-X1, Yokogawa Electric, Tokyo, Japan), as described previously [9], but with some modifications. The pipette solution for the Ca imaging contained the following (in mM): 135 potassium gluconate, 10 HEPES, 5 KCl, 5 NaCl, 5 Mg-ATP, 0.5 Na-GTP, 0.1 EGTA, 5 phosphocreatine, and 0.1 Oregon Green 488 BAPTA-1; pH 7.3. To optimize CF stimulation, the amplitude of CF-evoked EPSCs was maximized by adjusting the stimulus intensity and its location, as described above, in a voltage-clamp mode at -70 mV. Subsequently, the amplifier recording mode was switched to the current-clamp mode, which enabled the measurement of physiological Ca^{2+} responses. CF-evoked Ca^{2+} signals were acquired after setting the resting membrane potential of the recorded PCs to approximately -65 mV by current injection. Confocal fluorescence Ca^{2+} images were obtained at ~30 frames/s (33 ms exposure time, 512 × 512 pixels, no binning) with Andor iQ3 software (Andor, UK), and background-subtracted images were used for further analysis. F_b is the average basal fluorescence value during pre-stimulus frames (> 30 frames), and the relative Ca^{2+} signal change ($\Delta F/F_b$) was calculated pixel-by-pixel, where Ft is the fluorescence intensity

at time t and $\Delta F = Ft - F_b$. CF-evoked dendritic Ca^{2+} responses in PCs can vary depending on their dendritic location [10, 11]. Thus, to minimize the chance of missing or underestimating the Ca^{2+} signals, dozens of regions of interest (ROIs) (usually >20 ROIs) were set on the whole area of the active PC dendritic processes, and each ROI was smaller than the entire size of the dendritic Ca^{2+} signal spatial spread (Supplementary Fig. 5A) (9). The validity of the ROI position was routinely confirmed on averaged images of more than 20 frames (Supplementary Fig. 5, insets). The dendritic Ca^{2+} trace from each ROI was obtained as the time course of the spatially averaged values of $\Delta F/F_b$ within each ROI. The peak values, integrals, and half-decay times of the Ca^{2+} traces were measured from all the ROIs in each PC, and their maximum values were used to represent each PC [9]. Image processing and analysis were performed with Andor iQ3 (Andor, UK), and a set of custom-made macros or programs of ImageJ, Python, and Igor Pro 8 (WaveMetrics, USA) written by NH.

Western blotting

Samples from tissues of the cerebellar hemisphere were homogenized and sonicated with a lysis buffer (0.1 M Tris-HCl, 120 mM NaCl, 0.2% sodium deoxycholate, 0.8% NP-40, 2% SDS, 5% sucrose, 0.05% bromophenol blue, and 5% 2-mercaptoethanol). Homogenates were adjusted to 3 mg/ml using a sample buffer. The samples (10 μl) were loaded and separated by electrophoresis on 8% acrylamide gels and then transferred to polyvinylidene fluoride (PVDF) transfer membranes (iBlot 2 Transfer Stacks, PVDF, mini, Thermo Fisher Scientific). The membranes were washed with 20 mM Tris, 500 mM NaCl, and 0.05% Tween 20 (TBS-T) and blocked with a blocking solution (TBS-T with 5% skim milk) for 1 h. After washing with TBS-

T, the membranes were incubated with the following primary antibodies: rabbit anti-KCNMA1 (1:500; APC-107; Alomone Labs, Jerusalem, Israel) and mouse anti-β-actin (1:5000; A1978; Sigma Aldrich, St. Louis, MO) antibodies diluted in TBS-T overnight at 4 °C. After washing with TBS-T, the membranes were incubated with horseradish peroxidase-conjugated anti-rabbit IgG (1: 10000; Jackson ImmunoResearch Laboratories, West Grove, PA, USA) and HRP-conjugated anti-mouse IgG (1: 10000; Jackson ImmunoResearch Laboratories) diluted in TBS-T for 1 h at room temperature. After washing with TBS-T, the immunoblots were detected by chemiluminescence (Chemi-Lumi One L, Nacalai Tesque, Kyoto, Japan) with an imaging system (Fusion-solo s, Vilber-Lourmat, Collégien, France). The optical density of the protein bands was quantified using Evolution Capt software.

Statistical analysis

Significant differences were analyzed using Welch's t-test, Kruskal-Wallis test, one-way ANOVA, and two-way repeated-measures ANOVA followed by Bonferroni's post hoc test. Statistical analyses were performed using GraphPad Prism 7 (GraphPad Software, San Diego, CA, USA), R software statistical package (www.r-project.org), and SPSS software version 23. Data are expressed as the mean ± SEM.

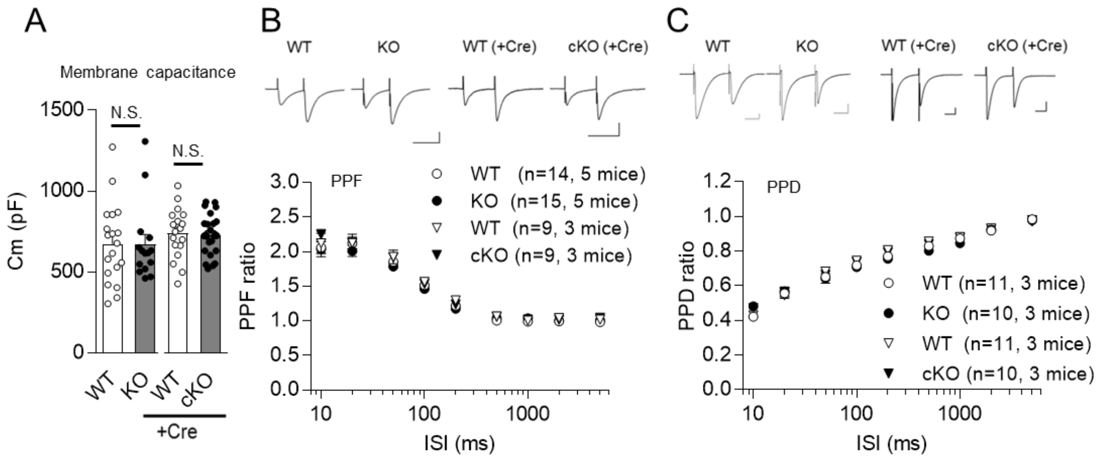

Supplementary Fig. 1. Systemic PKCγ-KO and cKO did not affect membrane capacitance and short-term synaptic plasticity in PCs

Whole-cell patch-clamp recordings were made from WT, PKCγ-KO, Cre-expressing WT, and Cre-expressing cKO (*Prkcg*$^{fl/fl}$) mouse PCs. **(A)** No significant difference in membrane capacitance between WT and PKCγ-deficient PCs., NS: not significant according to Welch's t-test. (WT; 674.8 ± 56.6 pF, n = 19 from 4 mice, KO; 672.4 ± 60.49 pF, n = 15 from 4 mice, *P* = 0.771, WT + Cre; 740.9 ± 36.82 pF, n = 18 from 4 mice, cKO; 731.2 ± 24.8 pF, n = 25 from 4 mice, *P* = 0.829). **(B, C)** Graphs showing the ratios of the PPF of PF-EPSCs **(B)** and PPD of CF-EPSCs **(C)**, which were determined based on the responses evoked by two stimuli with various interstimulus intervals. The PPF and PPD ratios were measured as the second EPSC amplitudes normalized to the first EPSC amplitude. The numbers of PCs and mice used are shown in the graphs. No significant difference was detected by repeated-measures ANOVA (WT vs. KO *P* = 0.636, WT + Cre vs. cKO *P* = 0.905, PPD; WT vs. KO *P* = 0.811, WT + Cre vs. cKO *P* = 0.736). Representative traces for stimulation with a 20-or 50 ms interstimulus interval are presented above the graph. Scale bars: 50 ms and 200 pA (B) or 20 ms and 200 pA (C). PKCγ, protein kinase

C γ isotype; PC, Purkinje cell; PPF, paired-pulse facilitation**;** PF-EPSCs, parallel fiber-evoked excitatory postsynaptic currents; PPD, paired-pulse depression; CF-EPSCs, climbing fiber-evoked excitatory postsynaptic currents.

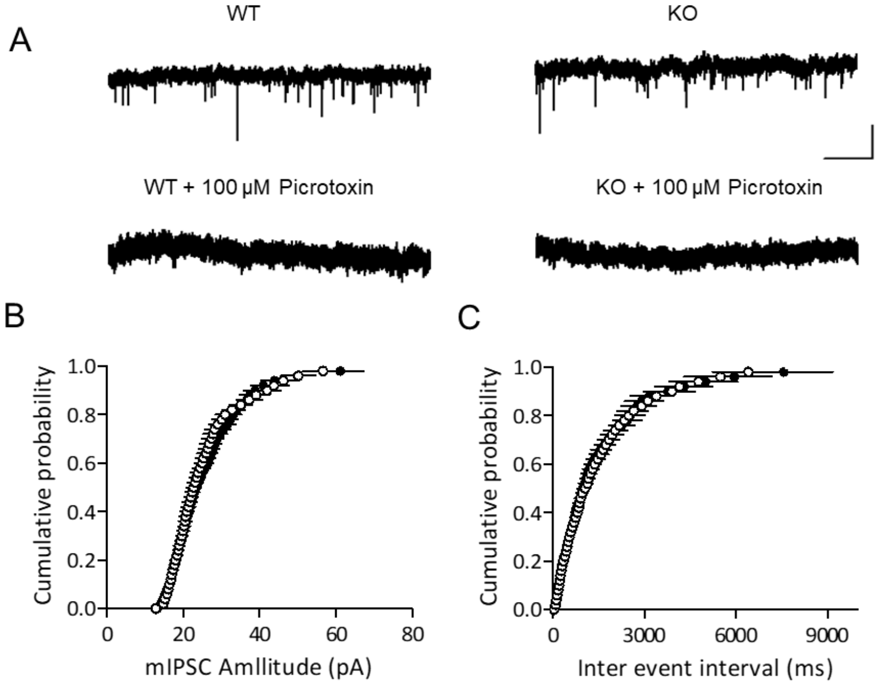

Supplementary Fig. 2. No significant difference in mIPSCs between WT and of PKCγ-KO mouse PCs

(A) Representative traces of mIPSCs recorded from WT (left) and KO (right) PCs. The currents were eliminated by the application of 100 μM picrotoxin to the extracellular medium. Scale bars: 20 ms and 400 pA. **(B)** Cumulative probability plots of mIPSC amplitudes. **(C)** Cumulative probability plots of mIPSC inter-event intervals. (WT: 7 cells from 4 mice, KO: 8 cells from 4 mouse). No significant change was detected using the Kolmogorov-Smirnov test (amplitude, P = 0.997; inter-event interval, P = 0.999). mIPSC; miniature inhibitory postsynaptic current, PKCγ; protein kinase C γ isotype, PC; Purkinje cell.

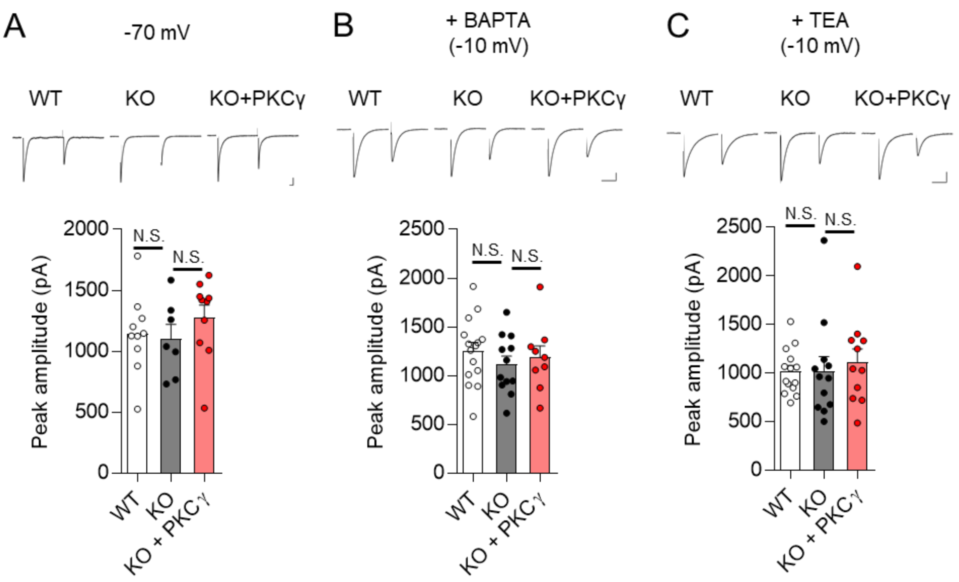

Supplementary Fig. 3. CF-EPSC amplitudes in PKCγ-KO mouse PCs were comparable to that in WT mouse PCs at -70 mV or in the presence of BAPTA or TEA

CF-EPSCs were recorded using a standard extracellular medium and an internal solution containing EGTA as a Ca^{2+} chelator from WT, PKCγ-KO, and rescued (KO + PKCγ) PCs. **(A)** CF-EPSCs recorded at -70 mV. **(B)** CF-EPSCs recorded at -10 mV, using an internal solution containing BAPTA instead of EGTA. **(C)** CF-EPSCs recorded at -10 mV in the presence of TEA, a K^+ channel blocker, in the extracellular medium. Representative CF-EPSC traces are shown in the graphs. Scale bars: 20 ms and 400 pA **(A)** or 20 ms and 200 pA. **(B, C)**. CF-EPSCs: climbing fiber-evoked excitatory postsynaptic currents, PKCγ; protein kinase C γ isotype; PCs, Purkinje cells; BAPTA, 1,2-bis-(o-aminophenoxy)-ethane-N,N,N',N'-tetraacetic acid; TEA, tetraethylammonium; EGTA, ethylene glycol-bis(β-aminoethyl ether)-N,N,N',N'-tetraacetic acid.

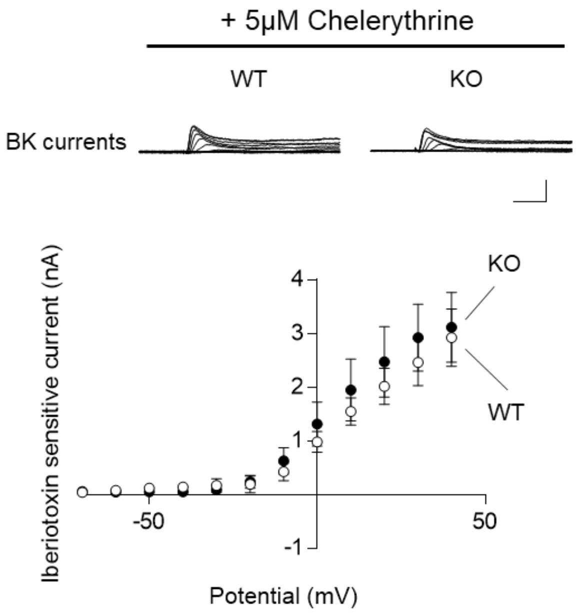

Supplementary Fig. 4. Incubation of cerebellar slices with a PKC inhibitor potentiated BK currents in WT mouse PCs.

Cerebellar slices from P17 WT and PKCγ-KO mice were perfused with an extracellular solution containing 5 μM chelerythrine, a membrane-permeable PKC inhibitor. Potassium currents were evoked in PCs at different membrane potentials from -70 mV to +40 mV in 10 mV increments in the extracellular solution without (control) or with 100 nM iberiotoxin. The net BK currents were calculated by subtracting the traces elicited with iberiotoxin from the control traces. The BK current sizes at peaks of fast components (2-5 ms after the current onset) are plotted in the graph. Representative traces of net BK currents are presented in the graph. Scale bars: 5 ms and 2 nA. Statistical analysis using repeated-measures ANOVA showed no significant difference in BK currents between the two genotypes. PKCγ, protein kinase C γ isotype; PCs, Purkinje cells; BK, Ca^{2+}-activated large-conductance K^{+}.

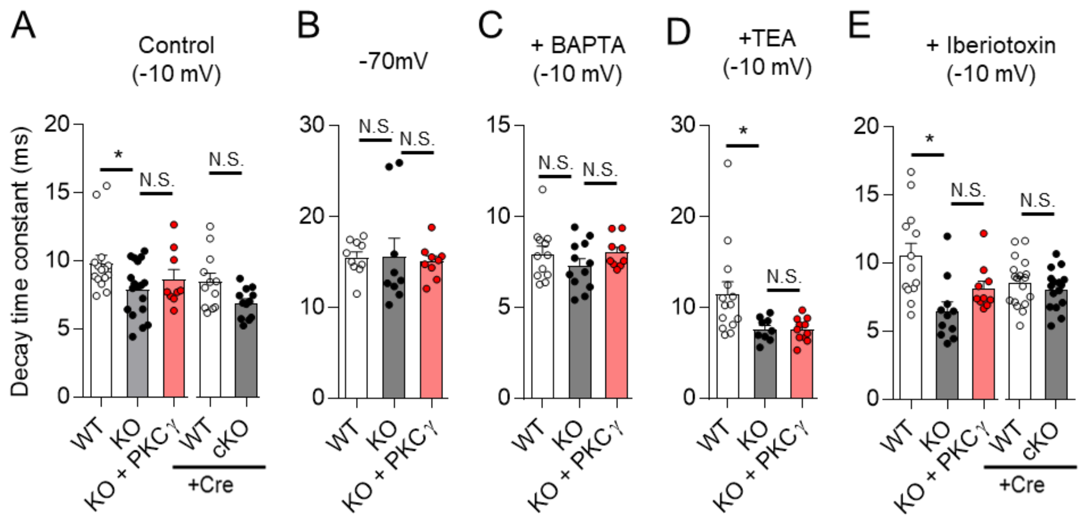

Supplementary Fig. 5. Faster decay time constants of CF-EPSCs in PKCγ-cKO mouse PCs were not restored by re-expression of PKCγ

Decay time constants were analyzed from the CF-EPSCs recorded in **Fig. 4** and **Supplementary Fig. 3**. **(A)** Decay time constants of CF-EPSCs recorded at -10 mV using a standard extracellular medium and an internal solution containing EGTA. **(B)** Decay time constants of CF-EPSCs recorded at -70 mV. **(C)** CF-EPSCs recorded at -10 mV using an internal solution containing BAPTA instead of EGTA. **(D)** CF-EPSCs recorded at -10 mV in the presence of TEA, a K^+ channel blocker, in the extracellular medium. **(E)** CF-EPSCs recorded at -10 mV in the presence of iberiotoxin, a BK channel blocker, in the extracellular medium. N.S., not significant; *$P < 0.05$, Welch's t-test or Bonferroni post-hoc test following one-way ANOVA. CF-EPSCs: climbing fiber-evoked excitatory postsynaptic currents; EGTA: ethylene glycol-bis(β-aminoethyl ether)-N,N,N',N'-tetraacetic acid; TEA, tetraethylammonium; BAPTA, 1,2-bis-(o-aminophenoxy)-ethane-N,N,N',N'-tetraacetic acid, BK; Ca^{2+}-activated large-conductance K^+.

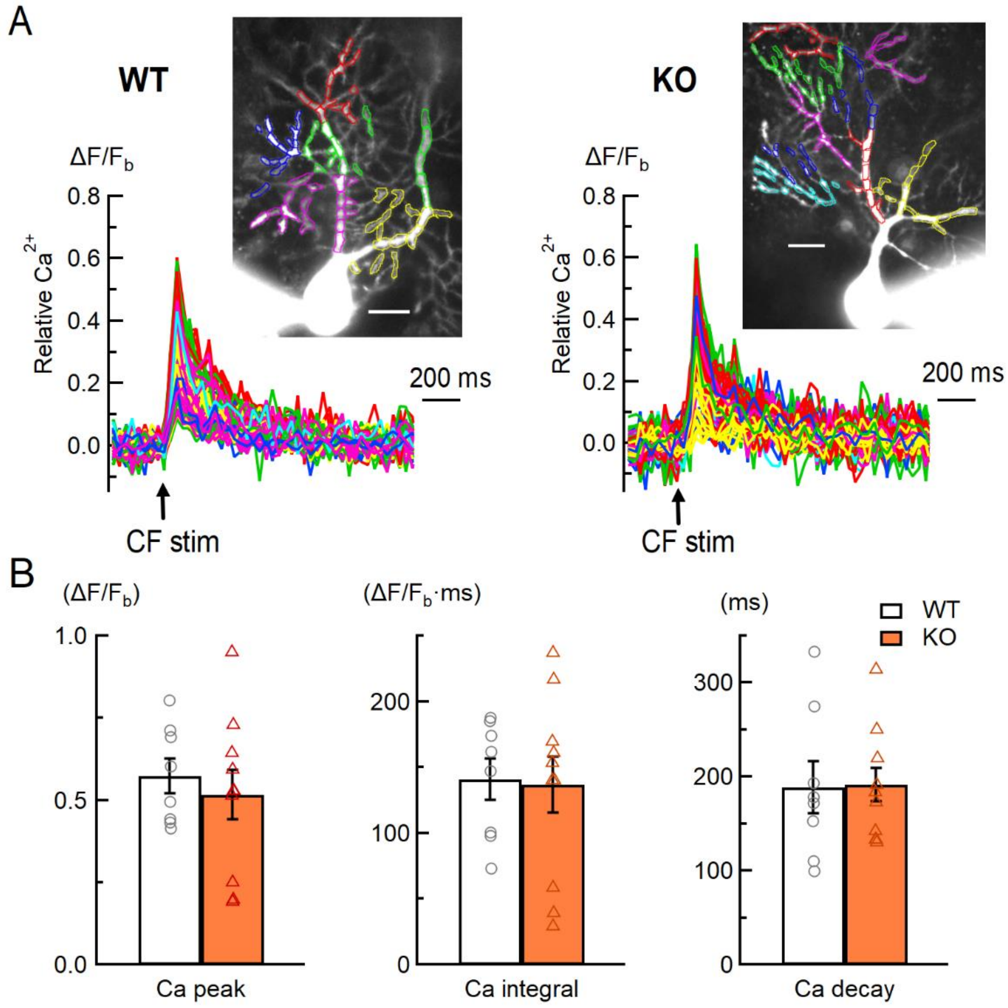

Supplementary Fig. 6. CF-evoked Ca^{2+} transients in PC dendrites were normal in PKCγ-KO mice

(A) Representative examples of CF-evoked Ca^{2+} signals in the PCs of WT (left) and PKCγ-KO (KO) mice (right). Inset images (scale bars, 20 μm) show ROIs on the active dendrites of the recorded PCs for analysis, and each Ca^{2+} trace ($\Delta F/F_b$ trace, see Methods) originates from the ROI with the same color code in each panel. Arrows indicate the time points of CF stimulation (CF stim). **(B)** Pooled data of the quantified Ca^{2+} signals (peaks, integrals, and half-decay times) in the WT (n = 8 from 4 mice) and PKCγ-KO mice (n = 10 from 4 mice). Bars and symbols indicate

the mean values of the data and the individual data points, respectively. There was no statistical difference in the Ca^{2+} signals between the WT and PKCγ-KO mice (peak, $P = 0.56$; integral, $P = 0.89$; half-decay time, $P = 0.94$). CF; climbing fiber, PC; Purkinje cell, PKCγ; protein kinase C γ isotype, ROIs; regions of interest.

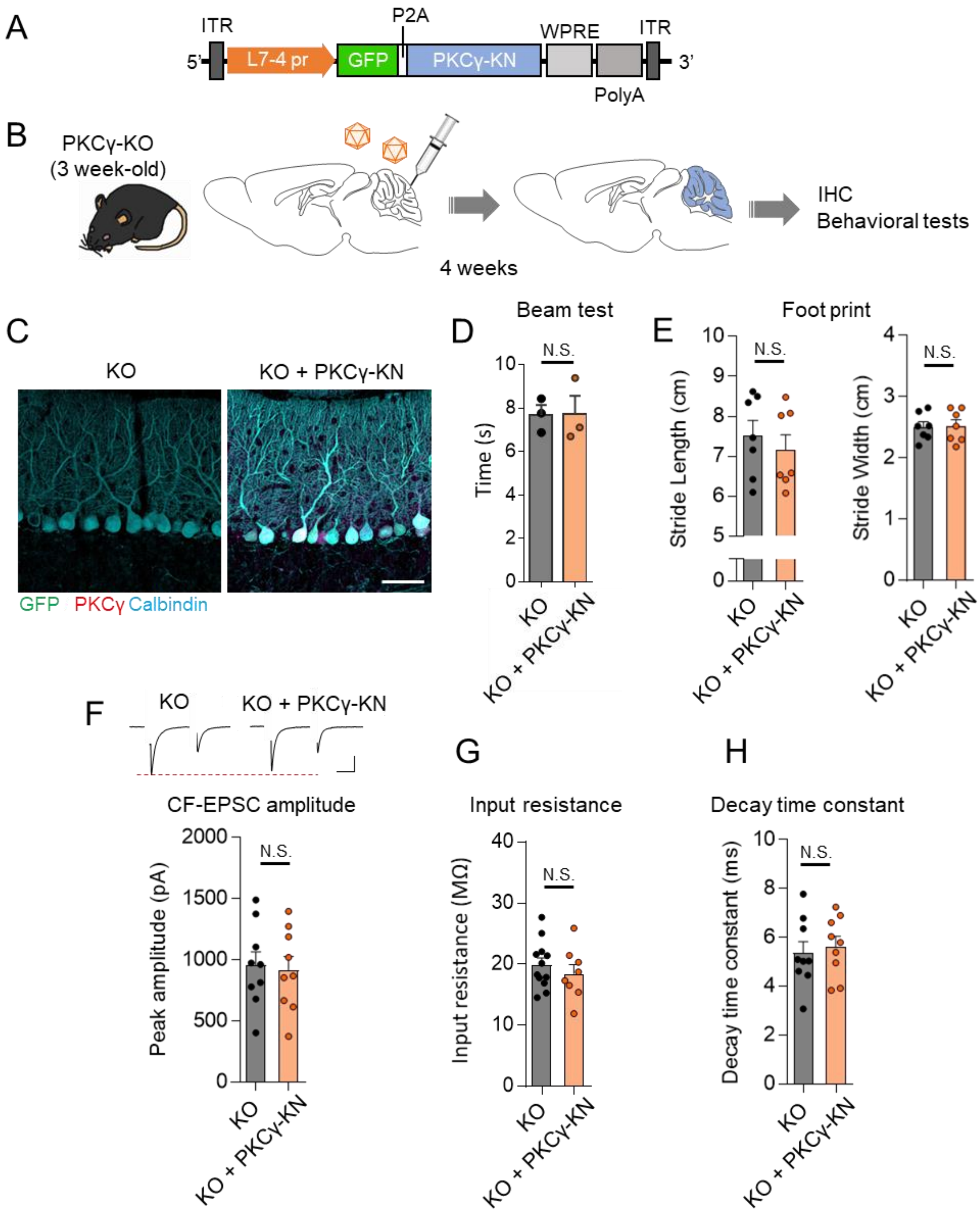

Supplementary Fig. 7. Kinase activity of PKCγ is indispensable to the rescue of aberrant phenotypes in PKCγ-KO mice

(A) Schema of AAV9 vectors expressing the kinase-negative mutant of PKCγ (PKCγ-KN) and GFP by a PC-specific L7-4-minCMV promoter. **(B)** Diagram depicting AAV vector-mediated PC-

specific expression of PKCγ-KN specifically in PKCγ-KO mice. Three-week-old PKCγ-KO mice received a cerebellar injection of the AAV9 vectors as illustrated in **(A)** at 1.6×10^{10} vg/mouse. The treated mice were examined using immunohistochemistry and behavioral tests 4 weeks after viral injection. **(C)** Immunohistochemistry of cerebellar sections from naïve PKCγ-KO (KO) and AAV-treated KO (KO + PKCγ-KN) mice. The sections were immunostained with antibodies against GFP (green), PKCγ (red), and calbindin (a marker for PC) (blue). Fluorescence signals were obtained using a confocal microscope. Scale bar, 100 μm. **(D, E)** Behavioral assessment of naïve PKCγ-KO (KO) and PKCγ-KN-expressing PKCγ-KO (KO + PKCγ-KN) mice by beam-walking test **(D)** and foot print analysis **(E)**. In beam-walking test, mice were walked on a 100-cm and φ1-cm steel bar and time spent on the 80-cm-walk was measured (both from 3 mice; N.S., not significant by Welch's t-test). In foot print analysis, stride lengths (left) and stride widths (right) are shown in the graphs (both from 7 mice; N.S., not significant by Welch's t-test). **(F-H)** Graphs showing the largest amplitudes of CF-EPSCs **(F)**, input resistances **(G)**, and decay time constants of CF-EPSCs **(H)** recorded from naïve and PKCγ-KN-expressing PKCγ-KO mouse PCs. Representative CF-EPSC traces are shown in graph **(F)**. Scale bars: 20 ms and 400 pA. NS, not significant according to Welch's t-test. PKCγ; protein kinase C γ isotype, PCs; Purkinje cells, GFP, enhanced green fluorescence protein; ITR, inverted terminal repeat; P2A, 2A peptide derived from porcine teschovirus-1, PolyA; polyadenylation signal sequence, L7-4 pr; L7-4 promoter with minimal cytomegalovirus sequence; WPRE, woodchuck hepatitis virus post-transcriptional regulatory element, AAV9; adeno-associated virus serotype 9, CF-EPSCs; climbing fiber-evoked excitatory postsynaptic currents.

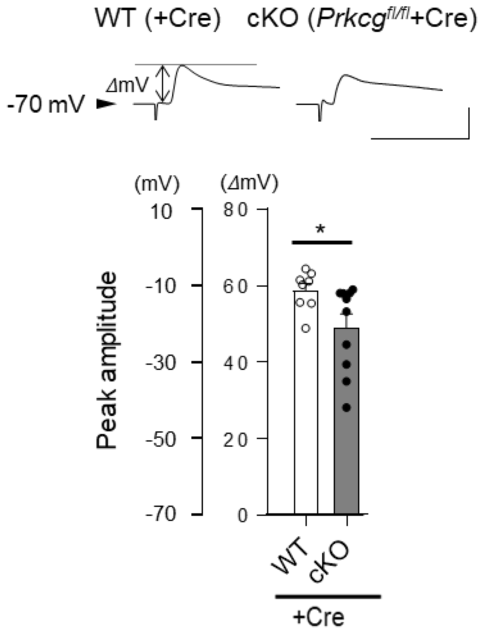

Supplementary Fig. 8. Significantly smaller amplitudes of CF-evoked depolarization recorded at PC soma from PKCγ-cKO mice than those from WT mice.

Whole-cell patch-clamp recordings were made from Cre-expressing WT and Cre-expressing cKO (*Prkcg*$^{fl/fl}$) mouse PCs. To selectively inhibit Na^+ spikes in PCs, the intracellular solution contained 1 mM QX-314, a membrane-impermeable voltage-gated Na^+ channel blocker. After the membrane potential of PC was adjusted to -70 mV by current injection, the granule cell layer was electrically stimulated to activate a CF innervating the recording PC, and the evoked depolarization was recorded at the soma. Membrane potential (mV) at the peak and the amplitude from the basal potential (-70 mV) to the peak of the depolarization (*Δ*mV) were plotted in the graph. Representative traces recorded at PC soma are shown above the graph. Scale bars: 10 ms and 50 mV. *$P < 0.05$, Welch's t-test. CF; climbing fiber, PC; Purkinje cell, PKCγ; protein kinase C γ isotype, cKO; conditional knock-out, WT; wild-type.

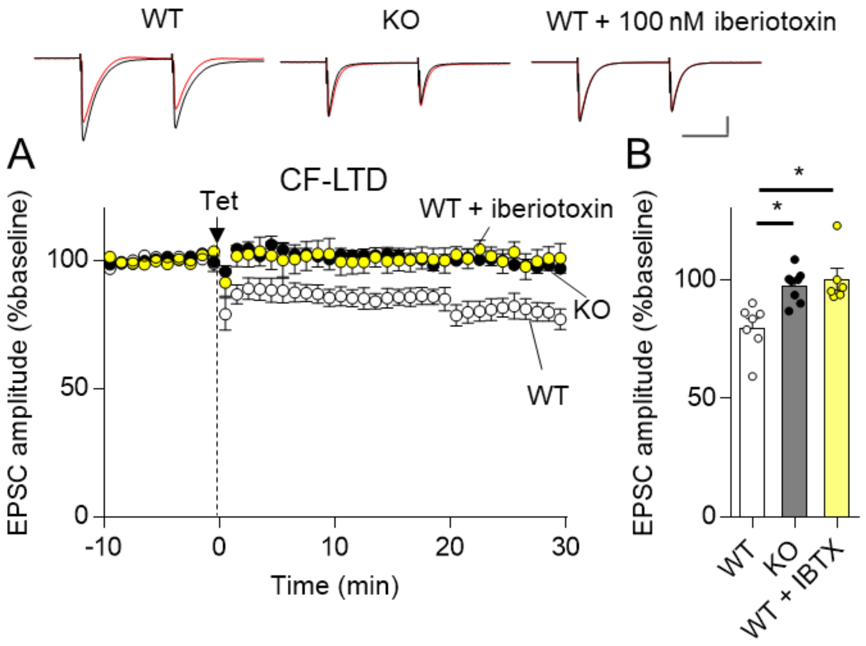

Supplementary Fig. 9. CF-LTD expression was impaired in PKCγ-KO mouse PCs or WT mouse PCs in the presence of iberiotoxin

(A) Time course of CF-EPSC amplitudes before and after tetanic stimulation (Tet) to CF (5 Hz, 30 s). Tetanic stimulation reliably induced CF-LTD in WT mouse PCs (WT; eight PCs from three mice), but not in PKCγ-KO mouse PCs (KO; 7 PCs from 3 mice) or WT mice PCs in the presence of 100 nM iberiotoxin in the extracellular medium (WT + iberiotoxin; six PCs from three mice). Representative traces before and 30 min after stimulation are shown as black and red lines, respectively. Scale bar: 50 ms, 400 pA. **(B)** Percentage ratios of CF-EPSC amplitudes 30 min after tetanic stimulation relative to those before stimulation. $^*P < 0.05$, Bonferroni post hoc test following one-way ANOVA. CF-LTD; climbing fiber long-term depression of synaptic transmission, PKCγ; protein kinase C γ isotype, PCs; Purkinje cells, CF-EPSCs; climbing fiber-evoked excitatory postsynaptic currents.

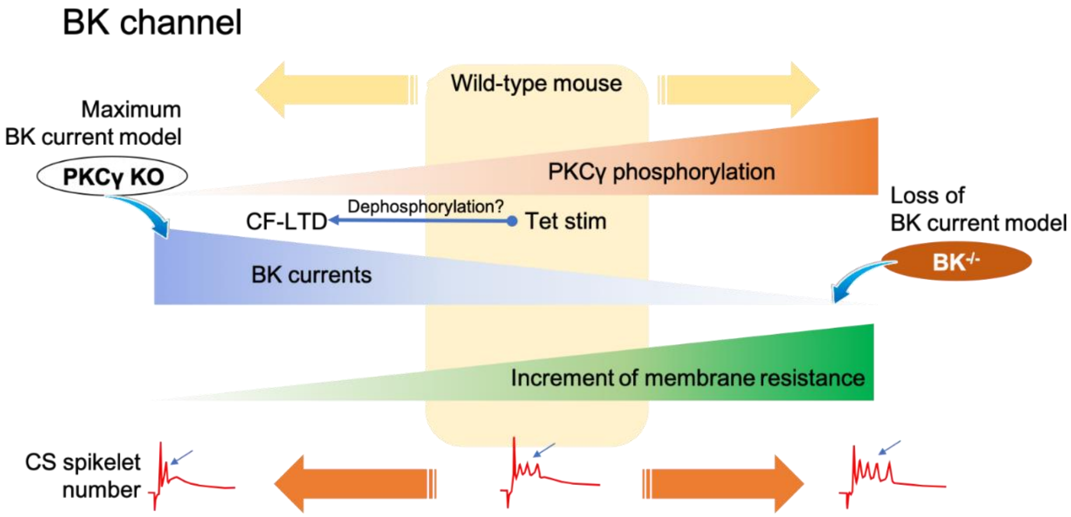

Supplementary Fig. 10. Schema depicting regulation of BK channel currents and CS waveform by PKCγ. BK channels in WT mouse PCs are moderately phosphorylated by PKCγ. Further phosphorylation by PKCγ (right yellow arrow) results in attenuation of BK currents with concurrent elevation of membrane resistance. Conversely, dephosphorylation (left yellow arrow) increases BK currents with reduced membrane resistance. Thus, $BK^{-/-}$ mice and PKCγ-KO mice serve as loss of BK current model and maximum BK current model, respectively. We assume that tetanic stimulation (Tet stim) to CF, which induces CF-LTD, dephosphorylates BK channels, resulting in potentiation of BK currents. Electrical signal decay along dendrites to soma in WT PCs is suppressed by further phosphorylation of BK channels (due to increased membrane resistance, right orange arrow), resulting in increased CS spikelet number (small arrow just above the CS trace). In contrast, dephosphorylation of BK channels (left orange arrow) potentiates the electrical signal decay and thus, results in decreased CS spikelet number. BK; Ca^{2+}-activated large-conductance K^{+}, CS; complex spike, PKCγ; protein kinase C γ isotype, WT; wild-type, PCs; Purkinje cells, $BK^{-/-}$; BK channel-knockout, KO; knockout, CF; climbing fiber; CF-LTD; climbing fiber long-term depression of synaptic transmission.

Supplementary Movie 1. Significant restoration of beam-walking performance in PKCγ-KO mice by PC-specific expression of PKCγ.

The wild-type mouse (WT) walked smoothly to the shelter with almost no slipping, whereas the PKCγ-knockout mouse (KO) wobbled on the bar with frequent slips, resulting in a significantly longer duration to reach the shelter. AAV vector-mediated PC-specific rescue of PKCγ in PKCγ-knockout mouse (KO + PKCγ) significantly improved their performance (fewer slips and shorter time to reach the shelter).

Supplementary Movie 2. Poor beam-walking performance in the *Prkcg*$^{fl/fl}$ mouse that lost PKCγ expression specifically from PCs after maturation.

The *Prkcg*$^{fl/fl}$ mouse received injection of AAV vectors expressing Cre by the PC-specific L7-6 promoter, resulting in PC-specific elimination of PKCγ expression (conditional knockout; cKO). In contrast to good performance of the wild-type mouse (WT), the cKO mouse showed significantly poorer performance, indicating that PKCγ expressed in mature mice PCs plays a critical role in motor coordination.

Supplementary Movie 3. PC-specific expression of kinase-negative mutant PKCγ failed to restore beam-walking performance in PKCγ-KO mice.

The PKCγ-knockout mouse (PKCγ-KO) at 7 weeks of age showed poor beam-walking performance. The poor motor performance was not restored by AAV-mediated PC-specific expression of kinase-negative mutant PKCγ (PKCγ-KO + AAV-PKCγ-(KN)), in contrast to significant rescue by AAV-mediated expression of wild type PKCγ **(Supplementary Movie 1)**.

SI References

1. Y. Matsuzaki, M. Oue, H. Hirai, Generation of a neurodegenerative disease mouse model using lentiviral vectors carrying an enhanced synapsin I promoter. *Journal of neuroscience methods* **223**, 133-143 (2014).
2. Y. Sawada, A. Konno, J. Nagaoka, H. Hirai, Inflammation-induced reversible switch of the neuron-specific enolase promoter from Purkinje neurons to Bergmann glia. *Scientific reports* **6**, 27758 (2016).
3. K. Nitta, Y. Matsuzaki, A. Konno, H. Hirai, Minimal Purkinje Cell-Specific PCP2/L7 Promoter Virally Available for Rodents and Non-human Primates. *Molecular therapy. Methods & clinical development* **6**, 159-170 (2017)
4. A. Iizuka *et al.*, Lentiviral vector-mediated rescue of motor behavior in spontaneously occurring hereditary ataxic mice. *Neurobiology of disease* **35**, 457-465 (2009).
5. M. Watanave *et al.*, Pharmacological enhancement of retinoid-related orphan receptor α function mitigates spinocerebellar ataxia type 3 pathology. *Neurobiology of disease* **121**, 263-273 (2019).
6. M. Watanave *et al.*, Contribution of Thyrotropin-Releasing Hormone to Cerebellar Long-Term Depression and Motor Learning. *Frontiers in cellular neuroscience* **12**, 490 (2018).
7. N. Takahashi *et al.*, Regulatory connection between the expression level of classical protein kinase C and pruning of climbing fibers from cerebellar Purkinje cells. *Journal of neurochemistry* 10.1111/jnc.14239 (2017).
8. C. Hansel, D. J. Linden, Long-term depression of the cerebellar climbing fiber--Purkinje neuron synapse. *Neuron* **26**, 473-482 (2000).
9. A. N. Shuvaev, N. Hosoi, Y. Sato, D. Yanagihara, H. Hirai, Progressive impairment of cerebellar mGluR signalling and its therapeutic potential for cerebellar ataxia in spinocerebellar ataxia type 1 model mice. *The Journal of physiology* **595**, 141-164 (2017).
10. A. Konnerth, J. Dreessen, G. J. Augustine, Brief dendritic calcium signals initiate long-lasting synaptic depression in cerebellar Purkinje cells. *Proceedings of the National Academy of Sciences of the United States of America* **89**, 7051-7055 (1992).
11. H. Miyakawa, V. Lev-Ram, N. Lasser-Ross, W. N. Ross, Calcium transients evoked by climbing fiber and parallel fiber synaptic inputs in guinea pig cerebellar Purkinje neurons. *Journal of neurophysiology* **68**, 1178-1189 (1992).
